# Quantifying generalization error in machine learning prediction of cognitive decline

**DOI:** 10.1016/j.tjpad.2026.100646

**Published:** 2026-08-08

**Authors:** Roya Melanie Hüppi, Nicolas Langer, Bruno Hebling Vieira, for the Alzheimer’s Disease Neuroimaging Initiative

**Affiliations:** a Methods of Plasticity Research, Department of Psychology, University of Zurich, 8050, Zurich, Zurich, Switzerland; b Neuroscience Center Zurich (ZNZ), University of Zurich & ETH Zurich, 8057, Zurich, Zurich, Switzerland; c Department of Adult Psychiatry and Psychotherapy, Psychiatric University Clinic Zurich and University of Zurich, 8032, Zurich, Zurich, Switzerland

**Keywords:** Cognitive decline, Structural MRI, Generalizability, Machine learning, Predictive modeling

## Abstract

**Background:**

Predicting cognitive decline as a continuum, from healthy age-related decline to mild cognitive impairment and dementia, enables more precise individual-level predictions. However, the practical value of such models for early intervention and prevention depends on their ability to generalize to independent cohorts, a property that is often not evaluated.

**Objectives:**

This study investigated whether adding structural magnetic resonance imaging (MRI) to non-brain data improved machine learning predictions of continuous cognitive decline and analyzed the models’ generalizability.

**Design:**

Multi-target random forest regression models predicted annual decline in the Clinical Dementia Rating Scale Sum of Boxes (CDR-SOB) and Mini-Mental State Examination (MMSE) using non-brain data, structural MRI data, or their combination from the Alzheimer's Disease Neuroimaging Initiative (ADNI; *N* = 1237) and Open Access Series of Imaging Studies (OASIS-3; *N* = 662) datasets. Cross-site generalizability was evaluated.

**Setting:**

Data from ADNI and OASIS-3 were used for this study.

**Participants:**

A total of 1899 participants who had demographic, clinical, and brain imaging data from a baseline session and clinical data from at least 2 follow-up sessions were included.

**Measurements:**

Baseline non-brain (demographics, clinical and neuropsychological scores, information on APOE genotype, cognitive diagnosis, health, and number of sessions before baseline) and/or structural MRI data were used to predict the yearly rate of change in CDR-SOB and MMSE scores.

**Results:**

Including structural MRI data improved prediction of CDR-SOB and MMSE change, reaching respective *R^2^* values of .41 and .33 in ADNI and .42 and .33 in OASIS-3. Model performance for across-dataset predictions was reduced (*R^2^* between .18 and .35), unexplained by distributional shifts of target variables. Models using only top predictive features performed similarly to full models when tested externally (*R^2^* between .18 and .34), suggesting predictor redundancy.

**Conclusions:**

Incorporating structural MRI data enhances within-dataset prediction of continuous cognitive decline, allowing for more precise individual-level prediction and advancing towards precision medicine. Even though external validation remains limited, quantifying the generalizability gap is a crucial step towards the responsible use of ML models in clinical intervention and prevention.

## Highlights

• Adding structural MRI to non-brain data improves prediction of cognitive decline.
• Cross-cohort evaluation reveals limits of model generalizability.
• Key-feature models match full-model performance in predictions across datasets.
• Continuous decline prediction captures interindividual variation in change.

## 1 Introduction

Pathological age-related cognitive decline, particularly due to Alzheimer’s disease (AD)-related neurodegeneration, affects executive functioning, memory, and language [1], causing substantial healthcare, economic, and social costs [2,3]. Early prediction is therefore critical, yet translating potential biomarkers of neurodegeneration, such as regional brain atrophy measurable by structural magnetic resonance imaging (MRI) [4], into actionable insights remains challenging. A key barrier is not only identifying accurate predictive markers but ensuring that such models are of practical utility by demonstrating robust generalizability across independent datasets and real-world clinical settings [5,6].

Machine learning (ML) offers a promising approach, enabling the joint analysis of large datasets, including imaging, genetic, clinical, lifestyle, and environmental data, to translate identified risk factors into diagnostic [[7], [8], [9], [10], [11]] and prognostic predictions [12,13]. However, many ML models are developed and evaluated within a single dataset, often relying on internal validation alone, leaving their generalizability to unseen, independently acquired data unclear [5,6]. This is particularly problematic in neuroimaging, where site-related heterogeneity can introduce bias and degrade model performance [14]. Additionally, ML models predicting cognitive decline often rely on the somewhat artificial diagnostic threshold between normal and pathological cognitive changes, providing a division into normal aging, mild cognitive impairment (MCI), and dementia [1]. To enable a more nuanced representation of the underlying process, cognitive decline can instead be studied as a continuum [15].

Following this continuous conceptualization and using data from the Open Access Series of Imaging Studies (OASIS-3), Vieira and colleagues [15] showed that combining structural MRI data with non-brain data improved prediction of continuous cognitive decline compared to non-brain data alone. However, while this work advanced predictive modeling, it did not examine the critical question of cross-dataset generalizability.

The present study addressed this gap by explicitly focusing on the generalizability of ML models for predicting continuous cognitive decline. Using data from the large, publicly available Alzheimer's Disease Neuroimaging Initiative (ADNI), we investigated whether including structural MRI data significantly improved prediction beyond non-brain data and, crucially, whether such models generalized across the independently acquired ADNI and OASIS-3 datasets. We compared within-dataset and cross-dataset predictive performance, evaluated generalizability across subgroups, examined post hoc target-distribution matching to mitigate bias, and assessed whether models based on a reduced feature set retained cross-dataset performance.

## 2 Methods

### 2.1 Study population and session selection

Data from two cohorts were included in this study. Relevant institutional ethics approvals were obtained in advance from the respective source institutions. The first dataset consisted of 662 participants from the OASIS-3 project. Data were provided by OASIS-3: Longitudinal Multimodal Neuroimaging (Principal Investigators: T. Benzinger, D. Marcus, J. Morris; NIH P30 AG066444, P50 AG00561, P30 NS09857781, P01 AG026276, P01 AG003991, R01 AG043434, UL1 TR000448, R01 EB009352). OASIS-3 is open-access longitudinal multimodal neuroimaging, clinical, and cognitive dataset for normal aging and Alzheimer’s disease from the Open Access Series of Imaging Studies (OASIS) stemming from various studies at the Washington University Knight Alzheimer Disease Research Center. The OASIS-3 dataset can be accessed at https://www.oasis-brains.org.

Baseline sessions (from clinical, neuropsychological, and MRI sessions) were used as input data, and follow-up clinical sessions, i.e., all clinical sessions after the baseline clinical sessions, were used as targets for the prediction of future continuous cognitive decline. To establish baseline data, MRI sessions were matched with their closest clinical session with a maximum absolute time difference of one year. Further, the closest neuropsychological session within one year of the MRI baseline session, when available, was selected as baseline data. However, baseline information from neuropsychological testing was regarded as optional, and the lack thereof was not a criterion for exclusion (see Treatment of Missing Data). No data prior to the selected baseline sessions were considered in the analysis. All baseline and follow-up clinical sessions were used to estimate the individual yearly rate of cognitive decline. To ensure a reliable estimation, participants needed at least three clinical sessions, i.e., one baseline and 2 or more follow-up sessions where both the Clinical Dementia Rating Scale Sum of Boxes (CDR-SOB) and the Mini-Mental State Examination (MMSE) were assessed. The final OASIS-3 sample included 662 participants.

For the second dataset, data were obtained from the ADNI database (adni.loni.usc.edu). The ADNI is led by Principal Investigator Michael W. Weiner, MD, and was launched in 2003 as a public-private partnership. The primary objective of ADNI has been to test whether serial MRI, positron emission tomography (PET), other biological markers, and clinical and neuropsychological assessment could be used jointly to measure the progression of MCI and early AD. For up-to-date information, see www.adni-info.org. To access the publicly and freely available ADNI data from the Laboratory of Neuro Imaging (LONI) Image and Data Archive (IDA) at https://adni.loni.usc.edu/data-samples/adni-data/, an application form including the investigator’s institutional affiliation and the proposed uses of the ADNI data must be submitted.

The procedure of the ADNI data selection was identical as described above for the OASIS-3 cohort. Consequently, 1237 ADNI participants that fulfilled inclusion criteria were included in this study (see Supplementary Material, Figure 1). Sample characteristics of the OASIS-3 and ADNI datasets are shown in Table 1. Chi-squared tests were performed to test for differences between datasets concerning sex and diagnoses, and *t*-tests were calculated regarding education, age, and the slopes of CDR-SOB and MMSE (α = .05).

**Table 1:** Sample characteristics of the OASIS-3 and ADNI datasets.

Characteristic	OASIS-3 (*N* = 662)			ADNI (*N* = 1237)		
	*M (SD)*	[min, max]	*n* missing	*M (SD)*	[min, max]	*n* missing
Demographics						
Age _baseline_	71.22 (8.24)	[46, 96]	–	71.54 (7.59)	[50, 94]	1
Sex	360 F; 302 M		–	595 F; 642 M		–
Years of education	15.74 (2.71)	[7, 29]	11	16.22 (2.63)	[7, 20]	40
Clinical scores						
MMSE _baseline_	28.31 (2.24)	[16, 30]	–	27.60 (2.59)	[13, 30]	–
CDR-SOB _baseline_	0.62 (1.37)	[0, 8]	–	1.38 (1.69)	[0, 11]	–
FAQ _baseline_	1.35 (3.38)	[0, 23]	14	2.71 (4.46)	[0, 26]	271
NPI-Q						
Presence _baseline_	0.95 (1.64)	[0, 10]	15	1.52 (1.95)	[0, 10]	245
Severity _baseline_	1.43 (2.78)	[0, 18]	15	–	–	245
GDS _baseline_	1.51 (1.99)	[0, 12]	17	1.38 (1.41)	[0, 7]	3
Genotyping (APOE alleles)	E2/E2: 5 E2/E3: 70 E2/E4: 21 E3/E3: 328 E3/E4: 202 E4/E4: 35		1	E2/E2: 1 E2/E3: 96 E2/E4: 23 E3/E3: 575 E3/E4: 409 E4/E4: 122		11
Diagnostic and sessions						
Clinical Diagnosis _baseline_	509 HC; 12 MCI; 111 DE*		30	454 HC; 607 MCI; 176 DE*		–
No. clinical sessions	5.8 (2.4)	[3, 15]	–	5.37 (2.37)	[3, 16]	–
Years between clinical sessions	1.2 (0.6)	[0.003, 5.5]	–	1.05 (0.61)	[0.00, 7.68]	–
Years in study	5.8 (2.5)	[1.6, 10.9]	–	5.36 (3.51)	[0.98, 16.48]	–
Outcomes						
MMSE slope (1/year)	–0.32 (0.93)	[–7.51, 2.94]	–	–0.67 (1.63)	[–13.72, 5.07]	–
CDR-SOB slope (1/year)	0.25 (0.61)	[–1.33, 4.42]	–	0.46 (0.96)	[–2.30, 8.18]	–

*Note. n* missing = number of participants with missing information on a particular variable; F = female; M = male; MMSE = Mini-Mental State Examination; CDR-SOB = Clinical Dementia Rating Scale Sum of Boxes; FAQ = Functional Activities Questionnaire; NPI-Q = Neuropsychiatric Inventory Questionnaire; GDS = Geriatric Depression Scale; APOE = apolipoprotein E; HC = healthy control; MCI = mild cognitive impairment; DE = dementia.

⁎ OASIS-3 included individuals with diverse kinds of dementia, whereas ADNI only included individuals with Alzheimer’s disease.

### 2.2 Predicting continuous cognitive decline

Non-brain data and structural MRI data were used to predict the yearly rate of future continuous cognitive decline. This decline was quantified using the trajectories of two clinical assessments, CDR-SOB and MMSE. The CDR-SOB captures impairment across six categories: memory, orientation, judgement and problem solving, community affairs, home and hobbies, and personal care, rated on a five-point scale where 0 represents no impairment and 3 corresponds to severe impairment. The sum score ranges from 0 to 18, with higher scores corresponding to higher impairment, and is suitable for the staging of dementia [16,17]. The MMSE, on the other hand, is widely used for screening and early detection of dementia [18], and includes the assessment of orientation, attention, memory, language, and the ability to copy a complex figure. The maximum score equals 30 and lower scores indicate more impairment [19,20].

Following the approach of Vieira and colleagues [15], an ordinary least squares linear regression model was fitted for each participant and for each target variable separately:

$${\hat{y}}_{i}=\beta_{0}+\beta_{1}\times X_{i}$$

where ${\hat{y}}_{i}$ is the estimated CDR-SOB or MMSE score of the follow-up clinical session, the intercept $\beta_{0}=y_{0}$ is the CDR-SOB or MMSE score at baseline and $X_{i}$ is the non-rounded number of years between the MRI session and the follow-up clinical session. $\;\beta_{1}$ is the resulting parameter which represents either the slope of CDR-SOB or MMSE change [15]. Both the slopes of CDR-SOB and MMSE change were predicted simultaneously, as multi-output models have previously demonstrated better generalizability to new cohorts than single-output models [21].

### 2.3 Non-brain data

Non-brain data features accounted for personal characteristics at baseline. Demographics included sex, age, and education. Clinical scores resulted from the CDR-SOB [17], MMSE [20], Functional Activities Questionnaire (FAQ) [22], Geriatric Depression Scale (GDS) [23] and Neuropsychiatric Inventory Questionnaire (NPI-Q) [24], and neuropsychological scores from the Boston Naming Test (BNT) [25], Trail Making Test (TMT) [26], word fluency, and Wechsler Memory Scale-Revised (WMS-R) [27]. APOE ε2, ε3, and ε4 allele counts (being either 0, 1, or 2) were included, accounting for genetic risk. Measures regarding cardio-and cerebrovascular health, diabetes, and hypercholesterolemia informed about participants’ health. The number of clinical sessions preceding the selected baseline session, i.e., sessions that could not be matched with an MRI session, were included to account for potential retest effects. Lastly, clinically conferred diagnostic classifications, either HC, MCI, or dementia, were part of the non-brain data features. See Supplementary Information and Table 1 in the Supplementary Material for more information.

### 2.4 Structural MRI data

For both datasets, the volume of grey matter regions, including accumbens, amygdala, caudate, hippocampus, pallidum, putamen, and thalamus, were extracted per hemisphere. For all these structures, decreasing volume over the course of AD has previously been observed [[28], [29], [30]]. Additionally, we included global measurements of total gray matter volume, total subcortical gray matter volume, left and right lateral, 3rd, and 4th ventricle volumes, left and right hemisphere mean cortical thickness, left and right total cortical volume, left and right cerebral white matter volume, left and right cerebellar white matter volume, left and right cerebellar cortical volume as well as the volume of five regions of the corpus callosum: anterior, mid-anterior, central, mid-posterior and posterior. Vieira et al. [15] have previously shown that this parsimonious feature set performs equally well in predicting cognitive decline as a more extensive set that includes 331 features from global, subcortical, and cortical (volume and thickness) markers. For details on the structural MRI data acquisition and preprocessing, see the Supplementary Information and Tables 1 and 2 in the Supplementary Material.

### 2.5 Treatment of missing data

Features with more than 25% missing data in either the ADNI or OASIS-3 dataset were excluded from both cohorts. In total, 18 non-brain features were excluded, reducing the total number from 66 to 48. Removing 10 features pertaining to neuropsychology test items, 4 features recapitulating family history of dementia and 4 features concerning smoking habits, lowered the number of neuropsychological features from 18 to 8 and the number of health information features from 17 to 9. No structural MRI features were excluded. After removing the features with excessive missing values, 4.0% of non-brain data was missing in OASIS-3 and 4.2% of non-brain data was missing in ADNI prior to imputation. Missing values where imputed with cross-validated iterative imputation [31]. This combination of multiple imputation with predictive modeling has been shown to work in different missingness scenarios [32].

### 2.6 Predictive analysis

The predictive model used in this study was a multi-target random forest (RF) regression model predicting the rate of decline of CDR-SOB and MMSE simultaneously. All predictive analyses were implemented in *Scikit-learn* 1.0.2 [31] in Python 3.9.13.

Predictive models were either trained and tested within the same dataset (*prediction within dataset*) or trained on one dataset and tested on the other (*prediction across datasets*). Three modalities were used for predictions within dataset: non-brain data only (*non-brain model*), structural MRI data only (*structural MRI model*), and the combination of non-brain data and structural MRI data (*combined model*). For predictions across datasets, a fourth modality was introduced: the *top 15 model*. This condition included only the 15 most predictive features of the respective training set, which were previously derived from analyzing the permutation importance of the non-brain features and structural MRI features for predictions within dataset (see Permutation Importance). As ML models can entail biases and may generalize poorly across different subgroups in the population [33], model performance of the combined model in predictions across datasets was evaluated across subgroups separated by diagnosis, sex, genetic risk (i.e., APOE ε4 allele count) and age. An additional analysis examined whether sign mismatches of the true versus predicted cognitive trajectories varied systematically across different diagnostic and sex-specific subgroups.

When training and testing within dataset, data was randomly split into 80% training and 20% test sets using ShuffleSplit. Splitting was repeated 1000 times. For predictions across datasets, the model was trained on the full sample of one dataset and tested on the full sample of the other, meaning that either the OASIS-3 dataset served as the training set and the ADNI dataset as the test set, or vice versa. Full datasets were used to obtain the best possible model. The step of evaluating the prediction capability of the models on independent test data leads to the determination of its generalization performance [34].

Since ADNI comprises a larger sample than OASIS-3, we performed additional analyses to isolate the contribution of sample size to cross-dataset performance estimates, by restricting the sample size to *n* = 60, 100, 200, 300, or 600 and repeating 100 times. In post hoc analyses, we further compared the multi-output RF model to the single-output model predicting CDR-SOB and MMSE separately, and the early to late fusion strategies. For the early fusion approach, the features from the two modalities were concatenated for prediction. For the late fusion strategy, separate models were first trained on non-brain and structural MRI features independently. The generated predictions were then combined using either *R²*-weighted averaging (where each modality's contribution is weighted by its predictive performance) or simple averaging.

### 2.7 Model evaluation

The models predicting within and across datasets were evaluated using the out-of-sample coefficient of determination (*R^2^*), the mean squared error (*MSE*), and the mean absolute error (*MAE*). For predictions within dataset, the evaluation metrics were calculated for each of the 1000 splits. For predictions across datasets, only one test set is available, therefore we measured and compared the distribution of the absolute error (*AE*), computed for each subject.

### 2.8 Model comparison

For model comparisons within the same dataset, the number of splits for which the model in question outperformed the reference model, according to *R^2^*, was calculated, resulting in a percentage value (see Vieira et al. [15]). Two-sided Wilcoxon signed-rank tests (α = .05) were performed to test for significance in differences in model performance. A two-sided Mann-Whitney U test (α = .05) was used to test differences in distributions of *R^2^* between models predicting within OASIS-3 and models predicting within ADNI. To compare the performance of two models predicting across datasets, the distributions of the *AE* were analyzed with a two-sided Mann-Whitney U test (α = .05). When comparing a model for prediction within dataset to a model for prediction across datasets, in a first step, the differences of *R^2^* were calculated for each split by using subtraction. Next, two-sided Wilcoxon signed-rank tests (α = .05) were performed with the adapted null hypothesis that mean difference is zero (zero_method = “wilcox”). To evaluate model performance in different subgroups, two-sided Mann-Whitney U tests (α = .05) for comparison of absolute errors were employed. The *p* values were corrected for multiple comparisons using the Holm-Bonferroni method.

### 2.9 Permutation importance

Permutation importance was calculated through permuting feature values to evaluate the influence of the features on the predictive performance of prediction within and across datasets. The difference in the predictive performance, as measured by the *R^2^* score, before and after the permutation of the predictive feature was used as a measure of importance. Hence, whenever the shuffling of a feature led to a performance decrease, the feature was deemed important for the model [35]. For the top 15 models, used only for predictions across datasets, the focus was on the 15 most predictive features of the respective training set. Importantly, the feature selection process was restricted to the training set, ensuring that no information from the test set influenced the selection, thereby avoiding circular analysis. The number of features was set at 15 due to the diminishing importance of additional features and based on prior work [13,15]. In a sensitivity analysis, this choice was compared with alternative feature set sizes of 5, 10, and 20 features.

To assess the joint contribution of correlated predictors, we additionally performed block permutation importance analyses [36]. This approach provides a more robust estimate of importance when predictors are strongly correlated, as the predictive information shared across features within a block cannot be compensated for by correlated variables that remain unpermuted. Rather than permuting individual features independently, predefined groups of related features were permuted simultaneously, thereby disrupting the information contained within the entire feature block while preserving the remaining predictors. Feature blocks were defined based on anatomical region, biological similarity or assessment type, and included, for example, hippocampal-amygdalar volumetric measures and APOE genotype variables (see Supplementary Material, Table 8 for full block definitions). Importance was quantified as the decrease in predictive performance (*R²*) following permutation of the entire block relative to the original model performance.

### 2.10 Code availability

The code is available on github.com/rhuepp/gemacode.

## 3 Results

### 3.1 Differences between the datasets

The sample characteristics of the two datasets are listed in Table 1. There were significant differences regarding sex (*Χ*^2^ = 6.555, *p* = .01), diagnoses (*Χ*^2^ = 440.064, *p* < .001), and education (*t*(1846) = 3.7, *p* < .001) between OASIS-3 and ADNI. In OASIS-3, there were proportionally more women, more HC and less individuals with MCI, and the participants had fewer years of education compared to the ADNI dataset. No significant difference was found concerning age (*t*(1896) = 0.9, *p* = .39). In addition, the expected yearly rate of change in CDR-SOB (*M_OASIS-__3_* = 0.25, *M_ADNI_* = 0.46, *t*(1897) = 5.9, *p* < .001) and MMSE (*M_OASIS-3_* = −0.32, *M_ADNI_* = −0.67, *t*(1897) = −6.1, *p* < .001) differed significantly between OASIS-3 and ADNI, with greater rates of decline being observed in ADNI. These differences are illustrated in the Supplementary Material, Figure 2, which displays the distribution of CDR-SOB and MMSE change in OASIS-3 and ADNI. Overall, the sample characteristics differed substantially between OASIS-3 and ADNI, illustrating realistically the typical variations observed across independent neuroimaging cohorts, and the importance of validation on independent datasets.

### 3.2 Prediction of continuous cognitive decline within dataset

**Fig. 1:**
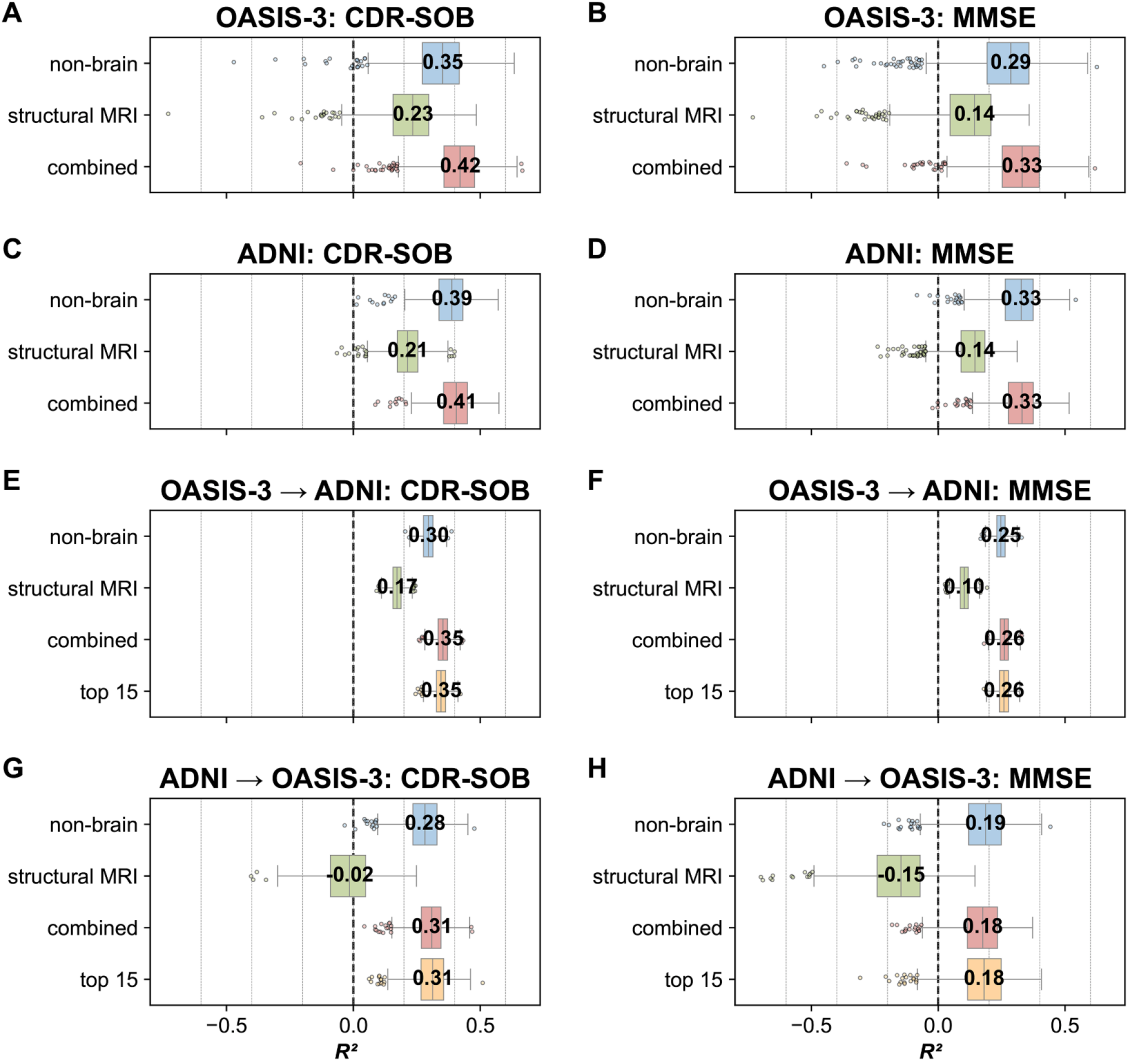
Model performance in OASIS-3 and ADNI.

*Note*. Model performance (*R^2^*, coefficient of determination; x-axis) for the input modalities (non-brain; structural MRI; combined, top 15; y-axis) to predict continuous cognitive decline measured via CDR-SOB and MMSE within and across datasets. For the predictions within dataset (A‒D), *R^2^* is measured across splits (*N*_splits_ = 1000), while for the predictions across datasets (E‒H), *R^2^* is measured across bootstrapped sample (*N*_boot_ = 1000). The number represents the median *R^2^* of the respective model. The dashed vertical line marks *R^2^* = 0.

The combined model led to the best prediction of continuous cognitive decline for both CDR-SOB and MMSE in OASIS-3 (CDR-SOB: *R^2^* = .42, *MSE* = .21, *MAE* = .24; MMSE: *R^2^* = .33, *MSE* = .56, *MAE* = .43; see Table 2, Table 3). The combined model significantly outperformed both the non-brain model (CDR-SOB: *R^2^* = .35, *MSE* = .24, *MAE* = .26; MMSE: *R^2^* = .29, *MSE* = .61, *MAE* = .44) in 88.8% of the splits for CDR-SOB, *p <* .001, and in 81.3% of the splits for MMSE, *p <* .001, and the structural MRI model (CDR-SOB: *R^2^* = .23, *MSE* = .28, *MAE* = .30; MMSE: *R^2^* = .14, *MSE* = .74, *MAE* = .53) in 97.2% of the splits for CDR-SOB, *p <* .001, and 96.3% of the splits for MMSE, *p <* .001. Additionally, structural MRI models performed significantly worse than non-brain models in 82.8% of the splits for CDR-SOB, *p <* .001, and in 87.8% for MMSE, *p <* .001. Performance for the prediction of CDR-SOB and MMSE slopes within OASIS-3 are shown in Fig. 1.A and Fig. 1.B, respectively.

**Table 2:** Model performance and model comparisons for predicting CDR-SOB change.

Dataset	Modality		*R^2^*	*MSE*	*MAE*	OASIS-3			ADNI			OASIS-3 → ADNI		ADNI → OASIS-3
						1	2	3	1	2	3	3	4	3
OASIS-3	1	non-brain	.35	.24	.26									
	2	structural MRI	.23	.28	.30	< .001*								
	3	combined	.42	.21	.24	< .001*	< .001*							
ADNI	1	non-brain	.39	.57	.42	< .001†								
	2	structural MRI	.21	.71	.52		< .001†		< .001*					
	3	combined	.41	.55	.41			.001†	< .001*	< .001*				
OASIS-3 → ADNI	1	non-brain	.30	.65	.50	< .001‡			< .001‡					
	2	structural MRI	.17	.77	.49		< .001‡			< .001‡				
	3	combined	.35	.60	.45			< .001‡			< .001‡			
	4	top 15	.34	.61	.42							< .001§		
ADNI → OASIS-3	1	non-brain	.28	.27	.29	< .001‡			< .001‡					
	2	structural MRI	−.02	.38	.41		< .001‡			< .001‡				
	3	combined	.31	.26	.30			< .001‡			< .001‡	< .001§		
	4	top 15	.31	.26	.27								<.001§	<.001§

*Note*. The model performance and the *p* values of model comparisons for predicting cognitive decline measured via Clinical Dementia Rating Scale Sum of Boxes (CDR-SOB) are displayed. OASIS-3 → ADNI = model trained on OASIS-3 and tested on ADNI; ADNI → OASIS-3 = model trained on ADNI and tested on OASIS-3. *R^2^* = median coefficient of determination across 1000 splits; *MSE* = average mean squared error across 1000 splits; *MAE* = average mean absolute error across 1000 splits.

⁎ Two-sided Wilcoxon signed-rank tests (α = .05) for comparison of *R^2^* (coefficient of determination).

† Two-sided Mann-Whitney U test (α = .05) for comparison of *R^2^*.

‡ Two-sided Wilcoxon signed-rank tests (α = .05) with the adapted null hypothesis for comparison of *R^2^*.

§ Two-sided Mann-Whitney U test (α = .05) for comparison of absolute errors.

**Table 3:** Model performance and model comparisons for predicting MMSE change.

Dataset	Modality		*R^2^*	*MSE*	*MAE*	OASIS-3			ADNI			OASIS-3 → ADNI		ADNI → OASIS-3
						1	2	3	1	2	3	3	4	3
OASIS-3	1	non-brain	.29	.61	.44									
	2	structural MRI	.14	.74	.53	< .001*								
	3	combined	.33	.56	.43	< .001*	< .001*							
ADNI	1	non-brain	.33	1.78	.76	< .001†								
	2	structural MRI	.14	2.24	.93		.201†		< .001*					
	3	combined	.33	1.76	.76			.999†	< .001*	< .001*				
OASIS-3 → ADNI	1	non-brain	.25	1.99	.81	< .001‡			< .001‡					
	2	structural MRI	.10	2.37	.89		< .001‡			< .001‡				
	3	combined	.26	1.95	.79			< .001‡			< .001‡			
	4	top 15	.26	1.96	.80							.233§		
ADNI → OASIS-3	1	non-brain	.19	.71	.48	< .001‡			< .001‡					
	2	structural MRI	−.15	1.00	.68		< .001‡			< .001‡				
	3	combined	.18	.72	.53			< .001‡			< .001‡	.001§		
	4	top 15	.18	.71	.47								< .001§	< .001§

*Note*. The model performance and the *p* values of model comparisons for predicting cognitive decline measured via Mini-Mental State Examination (MMSE) are displayed. OASIS-3 → ADNI = model trained on OASIS-3 and tested on ADNI; ADNI → OASIS-3 = model trained on ADNI and tested on OASIS-3. *R^2^* = median coefficient of determination across 1000 splits; *MSE* = average mean squared error across 1000 splits; *MAE* = average mean absolute error across 1000 splits.

⁎ Two-sided Wilcoxon signed-rank tests (α = .05) for comparison of *R^2^* (coefficient of determination).

† Two-sided Mann-Whitney U test (α = .05) for comparison of *R^2^*.

‡ Two-sided Wilcoxon signed-rank tests (α = .05) with the adapted null hypothesis for comparison of *R^2^*.

§ Two-sided Mann-Whitney U test (α = .05) for comparison of absolute errors.

Next, continuous cognitive decline was predicted within the ADNI dataset. In ADNI, adding structural MRI data to non-brain data led to a statistically significant improvement of the prediction for CDR-SOB (*R^2^* = .406, *MSE* = .55, *MAE* = .41, *p* = <.001) and MMSE (*R^2^* = .330, *MSE* = 1.76, *MAE* = .76, *p* = <.001), as compared to the non-brain model (CDR-SOB: *R^2^* = .388, *MSE* = .57, *MAE* = .42; MMSE: *R^2^* = .327, *MSE* = 1.78, *MAE* = .76). The combined model outperformed the non-brain model in 72.3% of the splits for CDR-SOB and 55.6% of the splits for MMSE, as well as the structural MRI model in 99.8% of the splits for CDR-SOB, *p <* .001, and 99.1% of the splits for MMSE, *p <* .001. Moreover, the structural MRI model (CDR-SOB: *R^2^* = .21, *MSE* = .71, *MAE* = .52; MMSE: *R^2^* = .14, *MSE* = 2.24, *MAE* = .93) performed worse than the non-brain model in 98.6% of the splits for CDR-SOB, *p <* .001, and 97.8% of the splits for MMSE, *p <* .001. Performance for the prediction of CDR-SOB and MMSE slopes within ADNI are shown in Fig. 1.C and Fig. 1.D, respectively.

Subsequently, the performance of models predicting continuous cognitive decline within the ADNI dataset and within the OASIS-3 dataset was compared by testing for differences regarding the distributions of *R^2^* between models. For histograms of *R^2^* for each split in all models see Supplementary Material, Figures 3 and 4. For the combined model, prediction within OASIS-3 was significantly better than within ADNI for CDR-SOB, *p* = .001, while for MMSE there was no significant difference to be found, *p* = .999. No significant differences in performance were found between ADNI and OASIS-3 when employing only structural MRI data or non-brain data in the prediction of both CDR-SOB and MMSE.

### 3.3 Prediction of continuous cognitive decline across datasets

To assess the generalizability of the model across datasets, models were first trained on the OASIS-3 dataset and subsequently evaluated on the independent ADNI dataset. The non-brain model had an *R^2^* = .30, *MSE* = .65, and *MAE* = .50 for CDR-SOB and an *R^2^* = .25, *MSE* = 1.99, and *MAE* = .81 for MMSE. The model performance of the structural MRI model was *R^2^* = .17, *MSE* = .77, and *MAE* = .49 for CDR-SOB and *R^2^* = .10, *MSE* = 2.37, and *MAE* = .89 for MMSE. Furthermore, the combined model demonstrated an *R^2^* = .35, *MSE* = .60, and *MAE* = .45 for CDR-SOB and an *R^2^* = .26 *MSE* = 1.95, and *MAE* = .79 for MMSE.

Likewise, models were then trained on ADNI and evaluated on OASIS-3. For the non-brain model, model performance was *R^2^* = .28, *MSE* = .27, and *MAE* = .29 for CDR-SOB and *R^2^* = .19, *MSE* = .71, and *MAE* = .48 for MMSE. The structural MRI model had an *R^2^* = −.02, *MSE* = 0.38, and *MAE* = .41 for CDR-SOB and an *R^2^* = −.15, *MSE* = 1.00, and *MAE* = .68 for MMSE. Finally, the combined model showed a performance of *R^2^* = .31, *MSE* = .26, and *MAE* = .30 for CDR-SOB and of *R^2^* = 0.18, *MSE* = 0.72, and *MAE* = 0.53 for MMSE. Supplementary Material, Figure 5 shows the true versus predicted values of CDR-SOB and MMSE change derived from combined models to predict continuous cognitive decline across datasets.

Model comparisons demonstrated that for the three modalities, i.e., non-brain, structural MRI, and combined, models predicting continuous cognitive decline within OASIS-3 were significantly better than models trained on OASIS-3 and tested on ADNI as well as models trained on ADNI and tested on OASIS-3 for both CDR-SOB and MMSE (*p <* .001 for each comparison). Likewise, models predicting continuous cognitive decline within ADNI significantly outperformed models trained on OASIS-3 and tested on ADNI as well as models trained on ADNI and tested on OASIS-3 for both CDR-SOB and MMSE for the three modalities (*p <* .001 for each comparison). The comparison of combined models predicting continuous cognitive decline across datasets showed that the model trained on OASIS-3 and tested on ADNI performed significantly better than the model trained on ADNI and tested on OASIS-3 for CDR-SOB, *p <* .001, and MMSE, *p =* .001. An overview of the model performance of all models and *p* values in model comparisons is provided in Table 2 for predicting CDR-SOB change and Table 3 for predicting MMSE change.

Additional analyses demonstrated that, as expected, *R^2^* increased with available sample size and plateaued before *n* = 600. The models trained on ADNI predicting MMSE in OASIS-3 attained smaller *R^2^* than the models trained on OASIS-3 and tested on ADNI across all training sample sizes, while no discernible differences were present for CDR-SOB. Further, single-output models did not achieve higher *R^2^* than multi-output models when trained on smaller subsets; however, a slightly superior performance existed when employing the full dataset for training (see Supplementary Material, Figure 7). In comparison to the early fusion strategy, both late fusion approaches resulted in a 4–17% decrease in *R²* for models trained on OASIS-3 and tested on ADNI. On the other hand, models trained on ADNI and tested on OASIS-3 benefited particularly from late fusion using simple averaging, with an increase in *R²* of 6–25% (see Supplementary Material, Table 5).

### 3.4 Evaluating model performance in different subgroups

**Fig. 2:**
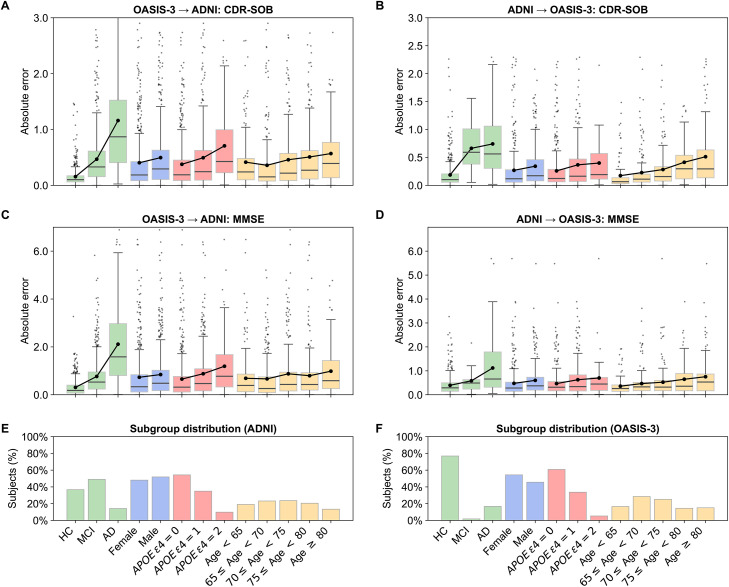
Distribution of absolute error across subgroups.

*Note*. Distribution of absolute error across subgroups in combined models predicting CDR-SOB and MMSE across datasets. Box plots represent the error distributions for CDR-SOB (A, B) and MMSE (C, D) when trained on OASIS-3 and tested on ADNI (A, C) and when trained on ADNI and tested on OASIS-3 (A, D). Dots connected by lines show the average absolute error. Bar plots visualize the distribution of the subgroups in ADNI (E) and OASIS-3 (F).

Considering a potential lack of generalizability and common biases in model predictions across subgroups, the combined models’ performance across datasets was assessed within subgroups defined by diagnosis, sex, APOE ε4 status, and age. Statistical testing revealed significant differences between males and females, individuals with MCI or dementia and normal controls, homozygous APOE ε4 carriers and heterozygous carriers or non-carriers, as well as different age groups (see Supplementary Material, Table 3). The *MAE* of predicting CDR-SOB and MMSE increases with age, cognitive impairment severity, and APOE ε4 allele dose, and is higher in males than females. Fig. 2 shows the *AE* distribution across subgroups.

The additional analysis of sign mismatches of the true versus predicted cognitive trajectories across different subgroups revealed that, overall, the models predicted trajectory direction well for HC and AD, with correct direction rates ranging from 82 to 97% for CDR-SOB. However, when the model was trained on OASIS-3 and tested on ADNI, 30% of MCI participants who truly improved were incorrectly predicted to worsen on CDR-SOB. For MMSE, correct direction rates were lower (∼77–82%) and errors in both mismatch directions occurred more frequently (see Supplementary Material, Table 6 and Figure 5). Across both predictive outcomes and cross-cohort prediction directions, males showed a slightly but consistently higher tendency (by 2.6–5.6%) to have true improvement incorrectly classified as worsening (see Supplementary Material, Table 7).

### 3.5 Most predictive features

Permutation importance was used to characterize the most predictive features of the best-performing modality, which was the combined model for both predictions within OASIS-3 and within ADNI. For OASIS-3, the baseline scores of the targets CDR-SOB and MMSE, word fluency, parts of the WMS-R and the TMT, as well as the sum score from the FAQ were part of the 15 most important features. From the structural MRI features, the volume of the left and right hippocampus and left and right amygdala along with the central corpus callosum, the volume of the right thalamus and the volume of the right nucleus accumbens were of importance.

In ADNI, the baseline score of the target feature MMSE, but not CDR-SOB, ranked among the 15 most important features. Moreover, word fluency, BNT, parts of the WMS-R and the TMT, chronological age at baseline, and scores from NPI-Q and FAQ were included among the most important features. Important structural MRI features were the volumes of the right hippocampus and 4th ventricle along with the left hemisphere mean cortical thickness. Taken together, ten features deemed important for predictions within OASIS-3 also emerged as important when the model trained on OASIS-3 data was applied to ADNI data. Similarly, 8 out of the 15 most important features for predictions within ADNI overlapped with those identified when the model trained on ADNI was applied to OASIS-3 data. Table 4 and Figure 6 of the Supplementary Material provide detailed information on feature importance values.

According to the additional block feature permutation analysis, model performance was primarily driven by neuropsychological data. Clinical variables, brain-derived features, and FAQ items ranked among the most important features for both ADNI and OASIS-3. In ADNI, other cerebral features exhibit similar importance to hippocampal-amygdalar volumes. In contrast, hippocampal-amygdalar volumes achieved higher importance and were among the most important brain-derived predictors in OASIS-3, whereas other cerebral feature blocks contributed little to predictive performance (see Supplementary Material, Table 8).

### 3.6 Prediction of cognitive decline with top 15 model across datasets

Given the potentially substantial information overlap among the original features, models using only the top 15 features from each training set were evaluated to test whether more parsimonious models could maintain predictive performance across datasets. New models including only the top 15 features of the respective training set were used for the prediction of continuous cognitive decline across datasets. To avoid circular analysis, we did not perform predictions within dataset using the top 15 models. The model trained on the top 15 features of OASIS-3 and tested on the ADNI dataset resulted in a model performance of *R^2^* = .34, *MSE* = .61, and *MAE* = .42 for CDR-SOB and *R^2^* = .26, *MSE* = 1.96, and *MAE* = .80 for MMSE. The model trained on the top 15 features of ADNI and tested on the OASIS-3 dataset achieved an *R^2^* = .31, *MSE* = .26, and *MAE* = .27 for CDR-SOB and *R^2^* = .18, *MSE* = .71, and *MAE* = .47 for MMSE. Note that the importance of the 15th feature, especially in ADNI, approached zero. A sensitivity analysis with alternative feature set sizes of 5, 10, and 20 features indicated that *R²* values largely stabilized at 15 features, with little to no improvement observed beyond this threshold (see Supplementary Material, Table 5).

Next, for predictions across datasets, top 15 models were compared to combined models by analyzing the difference in distributions of the *AE*. Model comparison showed that for models trained on OASIS-3 and tested on ADNI the top 15 model outperformed the combined model for CDR-SOB (*p* <.001), whereas for MMSE, there was no significant difference in performance (*p* = .23). When models were trained on ADNI and tested on OASIS-3, the top 15 model performed significantly better than the combined model for both CDR-SOB (*p* <.001) and MMSE (*p <* .001). Table 2, Table 3 provide an overview of the model performance of all models and display the *p* values of the model comparisons for models predicting CDR-SOB and MMSE change, respectively.

## 4 Discussion

This study investigated whether adding structural MRI to non-brain data improved the performance of an ML model predicting continuous cognitive decline and analyzed the models’ generalizability within and across datasets. We found that in ADNI, adding structural MRI to non-brain data significantly improved the prediction within dataset for both MMSE and CDR-SOB, although modestly for the latter. Predictions within one dataset (OASIS-3 or ADNI) were significantly and substantially better than predictions across datasets in any modality (see Figure 1; Table 2, Table 3). When models were reduced to a set of top predictive features, cross-dataset performance was retained.

Combined models achieved the highest predictive accuracy within each cohort, explaining up to 42% of the variance in CDR-SOB changes and 33% in MMSE changes. While non-brain data provided a strong baseline, structural neuroimaging contributed unique, additive value to the prognosis of cognitive trajectories. The identified performance improvement aligns with previous studies predicting cognitive decline categorically [7,12,13,37] and continuously [15]. The overall explained variance of the combined model was comparable to the *R²* of 0.34–0.42 reported by Vieira et al. [15] and slightly superior to the *R²* of 0.29 of a model predicting 2-year CDR-SOB change in amyloid beta positive AD individuals [38], though as shown in the subgroup analyses below, accurate prediction remains most challenging in the AD subgroup. However, this approach exhibited a generalizability gap when models were applied to independent datasets, with *R^2^* values decreasing by 17–45% relative to internal validation. This contrast between high internal consistency and reduced external validity highlights a pervasive challenge in ML applied to clinical data, where models frequently capture site-specific idiosyncratic patterns, such as variances in cohort composition or scanner-specific noise, rather than learning solely universally applicable biological features of cognitive decline [39]. Nevertheless, quantifying the generalizability gap in independently acquired data and characterizing its possible sources are crucial for translating research to real-world applications [39,40].

Overfitting constitutes a major challenge in ML [41], and larger sample sizes mitigate this risk. In our analyses, performance plateaus before *n* = 600 (see Supplementary Material, Figure 7), suggesting that model performance has stabilized within the sample sizes used in this study. Nevertheless, the high number of predictors, namely 48 non-brain and 35 structural MRI features, remains a potential source of overfitting [39]. To address this, we showed that the top-15 model performed comparably to the full model, with modest improvements in *MAE* that, while statistically significant, may not reflect clinically meaningful differences. This supports the use of parsimonious models in clinical practice, where readily obtainable predictors could achieve comparable prognostic performance while reducing data collection burden and improving feasibility in resource-constrained settings.

Although the composition of the top 15 features varied between OASIS-3 and ADNI, there was an overlap regarding the baseline score of MMSE, the sum score from the FAQ, word fluency, parts of the WMS-R and the TMT, and the volume of the right hippocampus. While it is somewhat evident for the baseline score of MMSE to be relevant, as it is included in the calculation of MMSE slope (see Modeling the Prediction of Continuous Cognitive Decline), past work has also shown that participants with lower MMSE scores are more likely to progress further towards dementia [42]. The baseline CDR-SOB score, however, was identified as one of the most important features of OASIS-3, but not of ADNI. This may result from the permutation approach underestimating the importance of correlated features, as shared information can remain when one correlated feature is permuted [15] (see Limitations for a more thorough exposition). The strong negative correlation between CDR-SOB and MMSE (i.e., in OASIS-3: *r*(660) = −0.64; in ADNI: *r*(1235) = −0.72), indicating that higher scores on the CDR-SOB are related to lower scores on the MMSE [43,44], could justify the baseline CDR-SOB score not falling within the top 15 features of ADNI. Moreover, previous research has shown that FAQ scores [45,46], category word fluency [47,48], WMS-R subtests [49], TMT performance [42,50,51], and hippocampal volume [52,53] can distinguish MCI from AD or predict dementia progression. For the cross-dataset models (see Supplementary Material, Figure 6), there was also substantial overlap in the most important features, including core measures of cognition (MMSE total), memory (WMS scores), executive ability (category word fluency), and medial temporal lobe structure (hippocampal and amygdalar volume), indicating their robust relevance for predicting cognitive decline. This suggests that routinely collected clinical, neuropsychological, and neuroimaging data may be sufficient to support individualized prognostic decisions across different clinical settings. This is particularly relevant for clinical trials, where MRI data are most often already collected to rule out comorbidity [54] and are sometimes used as an endpoint for neurodegeneration [55]. The relatively lower importance of the FAQ in models trained on ADNI and tested on OASIS-3 might have been influenced by dataset-specific missingness, with a large proportion of ADNI AD participants lacking this information. Differences in the exact ranking of features may also reflect cohort composition, with ADNI enriched for amnestic MCI and AD and OASIS-3 containing a larger proportion of cognitively normal participants from a more community-based sample.

Additionally, there are significant differences between the datasets, e.g., concerning the participants’ sex, diagnoses, and education, limiting the models’ generalizability. Therefore, model performance for predictions across datasets was compared for different subgroups (see Fig. 2). The combined model performed significantly better for HC than for individuals with MCI and AD in the respective test set (see Supplementary Material, Table 3), indicating reduced clinical utility and generalizability in more advanced disease stages. Notably, the present model's average MMSE *MAE* of 1.07 across MCI and AD individuals in external ADNI validation compares favorably to the 2.19 reported by Fogel et al. [56], who also validated externally on ADNI. However, this comparison should be interpreted cautiously, as that study relied solely on routinely collected clinical and demographic data, whereas the present model additionally incorporated neuropsychological scores, APOE genotype, and structural MRI.

In an additional analysis, the directions of true versus predicted cognitive trajectories were examined to assess whether slope sign mismatches varied systematically across subgroups (see Supplementary Material, Tables 6 and 7; and Figure 5). Overall, the models predicted trajectory direction well for HC and AD, but MCI represented a consistent exception: when the model was trained on OASIS-3 and tested on ADNI, almost a third of the MCI participants who showed true improvement were incorrectly predicted to worsen on CDR-SOB, indicating a systematic bias toward over-predicting decline in this diagnostic group. A similar, though less pronounced, pattern emerged for MMSE, with generally lower correct direction rates and more frequent errors in both mismatch directions, suggesting greater overall predictive uncertainty for this measure. These subgroup differences may partly be explained by the respective floor and ceiling effects in CDR-SOB and MMSE. 55.1% of OASIS-3 and 20.5% of ADNI participants have a baseline CDR-SOB equal to zero and no change over time. Most of these participants were HC: 99.7% in OASIS-3 and 100% in ADNI. This suggests that floor effects occurred largely due to HC, for whom cognitive decline is minimal or absent, facilitating their prediction for the models. 6.9% of OASIS-3 and 4.0% of ADNI participants showed a baseline MMSE equal to 30 as well as no change over time. Among these participants, 93.5% of OASIS-3 and 86% of ADNI were HC. While ceiling effects in MMSE were less impactful than floor effects in CDR-SOB, they may still have contributed to better model performance for HC in the test set.

Along with this, models predicting cognitive decline across datasets performed significantly better for APOE ε4 non-carriers than for participants carrying either one or two APOE ε4 alleles in three out of four comparisons (see Figure 2; Supplementary Material, Table 3). This could be a compound effect, since carrying at least one APOE ε4 allele is one of the strongest risk factors for sporadic AD [3]. Performance was also found to decrease with increasing age. Regarding sex, predictions for females were more accurate on average, a pattern also reflected in the slope sign mismatch analysis: males were slightly but consistently more likely to have true improvement misclassified as worsening across both outcomes and directions of cross-cohort predictions (see Supplementary Material, Table 7; and Figure 5). While these sex-based differences were modest in magnitude relative to the diagnosis-based differences, they suggest a subtle but consistent directional bias in male predictions.

The difference regarding participants’ diagnoses in OASIS-3 and ADNI further manifests as a significant difference in the decline rate of both CDR-SOB and MMSE, with greater slopes in ADNI. To address this, a post hoc analysis was performed, where the distribution of the predictive targets (CDR-SOB and MMSE slope) of the test set was matched to that of the training set in the prediction across datasets. This matching procedure led to an improvement concerning *AE* but an observable reduction in *R^2^* in all the comparisons of models trained on OASIS-3 and tested on ADNI. For models trained on ADNI and tested on OASIS-3, the matching procedure led to worsening regarding *AE* but mostly observable improvements in *R^2^* (see Supplementary Material, Table 5). This might be due to the model trained on OASIS-3 being more accurate for participants with less severe decline and, conversely, the model trained on ADNI performing better in participants with more severe impairment. Matching the test set to the training set distribution reduces the variance of the models’ outputs when trained on OASIS-3 and tested on ADNI. In models trained on ADNI and tested on OASIS-3, it increases the proportion of subjects with some level of impairment, which are usually linked to worse *AE*. Thus, we demonstrated that performance differences cannot be solely attributed to different distributions of target variables. Nevertheless, it cannot be ruled out that subsamples built based on matching other variables, such as sex, age, or ethnicity, would yield different results, as bias has been demonstrated to be a challenge there [33,[57], [58], [59], [60]].

### 4.1 Limitations and future research

Estimating cognitive decline as a continuous variable requires longitudinal data from various clinical assessments. We applied participant-specific linear models to estimate the target variables, since linear trajectories can robustly be estimated with only three measurements and approximate the immediate rate of change. However, more complex, non-linear trajectories of future CDR-SOB and MMSE might better represent cognitive decline [15,61], even though they require more longitudinal measurements per participant, and, while likely less biased, are more prone to variance.

ML algorithms are often deemed as “black boxes”, i.e., the mapping from inputs to outputs is uninterpretable, which potentially reduces the outputs’ usefulness [6]. To counteract this problem, feature importance was evaluated comparing *R^2^* scores before and after permuting each feature [5,35]. However, highly correlated features present a challenge for feature importance estimation [15], which might explain the previously mentioned omission of baseline CDR-SOB among highly ranked features in ADNI, or the low importance attribution of APOE ε4 allele count, despite being well-established as the highest genetic risk factor for AD [3], as it is negatively correlated with APOE ε2 and ε3 allele counts. Further, homotopic regions, such as the left- and right-hippocampus volumes, may, for example, encode partially shared biological signals, allowing models to rely on either feature, or a combination, which attenuates their permutation importance and possibly arbitrarily attributes higher permutation importance to one hemisphere. If one hemisphere-specific feature is selected earlier in the construction of an RF tree split structure, subsequent splits may not benefit from including the corresponding contralateral feature, since the available predictive signal is already largely captured. To better capture the joint contribution of correlated feature groups and provide a more stable assessment of predictive value at the level of biological features, anatomical systems, or assessment types rather than individual variables, block feature importance can be used. Using this strategy, we found that jointly permuting APOE features did not lead to appreciable performance degradation, suggesting that, on average, models do not rely on these features. Moreover, jointly permuted hippocampal-amygdalar volumes were more important than cortical features in OASIS-3, but not in ADNI, while, in isolation, no cortical feature attained high importance (see Supplementary Material, Tables 4 and 8). Importantly, feature importance reflects predictive contribution under the model’s learned representation and feature interactions, rather than causality. Therefore, high predictive utility does not necessarily imply causal relevance to the neurodegenerative process and, conversely, causal drivers of cognitive decline do not need to rank high in permutation importance.

The number of predictors to be included in the restricted model was somewhat artificially set at 15 based on prior work [13,15] and due to diminishing importance of additional features. A sensitivity analysis comparing this choice with alternative feature set sizes of 5, 10, and 20 features showed that model performance largely plateaued at 15 features, with *R²* values stagnating beyond this point (see Supplementary Material, Table 5). Furthermore, the multi-output models employed in this study performed comparably to single-output models examined in an additional analysis, except when trained on the full dataset, where a marginal performance advantage of single-output models was observed (see Supplementary Material, Figure 7). Future studies may therefore consider both approaches.

When combining non-brain and structural MRI data to predict cognitive decline, this study followed an early fusion approach as established in earlier work [15]. In a post hoc analysis (see Supplementary Material, Table 5), this approach was compared with the late fusion strategy. For models trained on OASIS-3 and tested on ADNI, both late fusion approaches resulted in a 4–17% decrease in *R²*. However, models trained on ADNI and tested on OASIS-3 benefited particularly from late fusion using simple averaging, with an increase in *R²* of 6–25%. Future studies should therefore consider both early and late fusion strategies and additionally explore the potential and generalizability of deep learning-based approaches [62] in which richer MRI representations, such as transformer-derived embeddings, are fused with clinical predictors, as these two modalities have been shown to capture complementary aspects of AD progression [63].

We demonstrated significant performance differences for subgroups defined by diagnosis, sex, APOE ε4, and age, potentially limiting usage of such models in clinical settings. We additionally showed that a bias mitigation strategy based on matching target distributions could potentially worsen performance. Ultimately, ethical use of ML models in medicine should favor the highest accuracy across each subgroup, not necessarily enforcing performance parity across subgroups [64]. This is crucial for developing robust models that attain maximum performance in diverse populations and conditions.

Furthermore, uncertainty estimation is crucial for incorporation of ML models in clinical research and personalized medicine. While our within-dataset resampling approach and analysis of across-dataset performance variability allow estimation of uncertainty in population-level performance and provide insight into cross-cohort generalizability, our models are deterministic and provide point-estimates of cognitive decline. Individualized estimation of aleatoric uncertainty, the intrinsic variability of cognitive trajectories that cannot be reduced by collecting additional data, and epistemic uncertainty, the uncertainty due to finite training data, model misspecification, or limited coverage of the feature space, requires models capable of estimating distributions over outcomes and approaches that account for uncertainty in the model itself and its parameters, respectively [65]. Further research is needed to understand the extent to which these sources of uncertainty impact cross-cohort generalizability under distributional shifts.

### 4.2 Conclusion

This study supports evidence for the benefits of adding structural MRI to non-brain data in future cognitive decline prediction using two large-scale datasets. When assessing the performance of different models in independent datasets, we attested significant performance reduction in all conditions. Performance degradation in predictions across datasets could not be circumvented by matching the distributions of the target variables of the test set to those of the respective training set. Models trained only on the top 15 features of the respective training set performed virtually equal to models trained on all available features when tested externally, indicating that routine neuropsychological, clinical, and neuroimaging data may suffice for personalized prognosis across clinical settings. Our results quantify the generalizability gap in real-world applications of ML models, highlighting the importance of responsible and informed clinical use of ML in cognitive impairment and other areas [6,39]. Moreover, our investigation of cognitive decline as a continuous variable enables more precise predictions on an individual level and contributes to developments in the direction of precision medicine [15,66].

## Funding

This work was supported by the URPP “Dynamics of Healthy Aging”, by the Swiss National Science Foundation [10001C_197480], and by the UZH Postdoc Grant, grant no. [FK-23-086] at the University of Zurich. The sponsors had no role in the design and conduct of the study; in the collection, analysis, and interpretation of data; in the preparation of the manuscript; or in the review or approval of the manuscript.

## Consent statement

This study was conducted using publicly available, de-identified data from the OASIS-3 and ADNI cohorts. All participants in both studies provided informed consent, and data collection was approved by the respective local institutional review boards. The use of these datasets complies with their data use agreements and ethical guidelines.

## Data availability statement

ADNI data can be obtained from https://adni.loni.usc.edu/, OASIS-3 data from https://www.oasis-brains.org.

## Declaration of the use of generative AI and AI-assisted technologies in scientific writing and in figures, images and artwork

During the preparation of this work, the authors used ChatGPT and Claude to improve language and assist with coding. After using it, the authors reviewed and confirmed the content as needed and take full responsibility for the content of the publication.

## CRediT authorship contribution statement

**Roya Melanie Hüppi:** Conceptualization; formal analysis; methodology; project administration; software; visualization; writing – original draft (lead); writing – review and editing (equal). **Nicolas Langer:** Conceptualization; funding acquisition; resources; supervision; writing – review and editing (equal). **Bruno Hebling Vieira:** Conceptualization; data curation; formal analysis; funding acquisition; methodology; software; supervision; visualization; writing – original draft (supporting); writing – review and editing (equal).

## Declaration of competing interest

The authors declare that they have no known competing financial interests or personal relationships that could have appeared to influence the work reported in this paper.

## Appendix Supplementary materials

Image, application 1
