# Supplementary material for: Quantifying generalization error in machine learning prediction of cognitive decline
Source: J Prev Alzheimers Dis. 2026 Aug 8;13(9):100646. doi: 10.1016/j.tjpad.2026.100646 (PMC13476611; doi:10.1016/j.tjpad.2026.100646)
Supplement: Supplementary file 1 [file mmc1.docx]

**Quantifying generalization error in machine learning prediction of cognitive decline**

**Roya Melanie Hüppi^a, b, c, ✉^, Nicolas Langer^a, b^, Bruno Hebling Vieira^a, b, ✉^, for the Alzheimer’s Disease Neuroimaging Initiative***

^a^	Methods of Plasticity Research, Department of Psychology, University of Zurich, 8050 Zurich, Zurich, Switzerland

^b^	Neuroscience Center Zurich (ZNZ), University of Zurich & ETH Zurich, 8057 Zurich, Zurich, Switzerland

^c^	Department of Adult Psychiatry and Psychotherapy, Psychiatric University Clinic Zurich and University of Zurich, 8032 Zurich, Zurich, Switzerland

^✉^	Corresponding authors: Roya Melanie Hüppi (Lenggstrasse 31, 8032 Zurich, Zurich, Switzerland, [roya.hueppi@bli.uzh.ch](mailto:roya.hueppi@bli.uzh.ch)); Bruno Hebling Vieira (Binzmühlestrasse 14, 8050 Zurich, Zurich, Switzerland, [bruno.heblingvieira@uzh.ch](mailto:bruno.heblingvieira@uzh.ch))

*	Data used in preparation of this article were obtained from the Alzheimer’s Disease Neuroimaging Initiative (ADNI) database ([adni.loni.usc.edu](https://adni.loni.usc.edu/)). As such, the investigators within the ADNI contributed to the design and implementation of ADNI and/or provided data but did not participate in analysis or writing of this report. A complete listing of ADNI investigators can be found at: <http://adni.loni.usc.edu/wp-content/uploads/how_to_apply/ADNI_Acknowledgement_List.pdf>.

a. Supplementary Information

##### Non-Brain Data

Non-brain data incorporated diverse measures to describe personal characteristics at baseline. Demographics included sex, age, and education. Being a woman, advanced age, and having less education have been previously described as risk factors for Alzheimer’s disease (AD) [1–4]. Similar risk factors also exist for the less prevalent non-AD dementias. [5] Non-brain data further contained clinical scores from the Clinical Dementia Rating Scale Sum of Boxes (CDR-SOB; 6), Mini-Mental State Examination (MMSE; 7), Functional Activities Questionnaire (FAQ; 8), Geriatric Depression Scale (GDS; 9) and Neuropsychiatric Inventory Questionnaire (NPI-Q; 10). While the CDR-SOB and the MMSE are often used instruments for screening and to stage the severity of AD [11,12], the FAQ is commonly used to assess instrumental activities of daily living, for example, paying bills or remembering appointments, and therefore to distinguish individuals with AD from individuals with mild cognitive impairment (MCI) and healthy controls (HC) [13]. In addition, FAQ scores were found to predict progression from MCI to AD in ADNI data [14]. In line with research postulating neuropsychiatric symptoms, among them depressive symptoms, as risk factors for developing dementia [15,16], NPI-Q scores as well as GDS scores have shown predictive value for predicting conversion from MCI to AD [17,18].

Moreover, neuropsychological scores from the Boston Naming Test (BNT; 19), Trail Making Test (TMT; 20), word fluency, and Wechsler Memory Scale-Revised (WMS-R; 21) were part of the non-brain data. In previous research, all neuropsychological tests included have been associated with cognitive decline. The BNT, a widely used confrontation naming test [22,23], was demonstrated to be a predictor of conversion from MCI to AD [24]. Likewise, the TMT, measuring processing speed, mental flexibility, sequencing, and visual-motor skills [25,26], predicted conversion from MCI to AD [27,28] and dementia progression [29]. Word fluency, a measurement involving executive functioning and language processing [30,31], was used to distinguish individuals with MCI and AD [32] as well as to predict the risk of incident MCI [33]. Subtests of the WMS-R, providing an estimate of overall memory functioning [21], were argued to be predictors for differentiating between people with dementia and HC [34].

APOE ε2, ε3, and ε4 allele counts (being either 0, 1, or 2) were also incorporated in the non-brain data. Carrying at least one APOE ε4 allele is one of the strongest risk factors for AD, while APOE ε2 represents a potential protective genetic variant associated with a lower risk of AD compared with the neutral, and most prevalent, third variant, APOE ε3 [1,35,36]. Evidence also implicates APOE in non-AD dementias, such as vascular cognitive impairment [5] and dementia with Lewy’s bodies [37].

Clinically conferred diagnostic classifications, either HC, MCI, or dementia, were included as part of the non-brain data features. Individuals with MCI have previously been found to demonstrate a higher rate of cognitive decline than HC, but a slower decline than individuals with mild AD in longitudinal data [38]. Accounting for the health information of the participants, measures regarding cardio-and cerebrovascular health, diabetes, and hypercholesterolemia were included as well, as they all constitute risk factors for AD [15]. Finally, to account for potential retest effects, the number of clinical sessions preceding the selected baseline session, i.e., sessions that could not be matched with an MRI session, was included as non-brain data.

##### Structural MRI Data Acquisition

In the OASIS-3 study, MRI data were acquired on Siemens 3T scanners. The most predominantly used parameter combination was voxel size = 1 × 1 × 1 mm^3^, repetition time (TR) = 2400 ms, and echo time (TE) = 3 ms. In ADNI, 3T MRI data were acquired on scanners from three different vendors: Siemens, Philips, and GE HealthCare. The two most frequently used parameter combinations were voxel size = 1.2 × 1 × 1 mm^3^, TR = 2300 ms, and TE = 3 ms as well as voxel size = 1 × 1 × 1 mm^3^, TR = 2300 ms, and TE = 3 ms. 1.5T MRI scans from ADNI were not used. Further information about MRI data acquisition is displayed in the [Supplementary Material, Table 2](https://osf.io/up65f/files/osfstorage).

##### MRI Preprocessing

MRI data was preprocessed using *fMRIPrep* 1.4.1 in OASIS-3 and *sMRIPprep* 0.9.2 in ADNI (RRID: SCR_016216; 42,43), which are based on *Nipype* 1.2.0 (OASIS-3) and *Nipype* 1.8.1 (ADNI) (RRID: SCR_002502; 44–46). Correction for intensity non-uniformity (INU) of T1-weighted (T1w) images was performed using ‘N4BiasFieldCorrection’ [47]. T1w images were distributed with ANTs 2.3.3 (RRID: SCR_004757; 48), and used as the T1w-reference throughout the workflow. Skull-stripping of each T1w-reference was applied with a *Nipype* implementation of the ‘antsBrainExtraction.sh’ workflow from ANTs, having OASIS-30ANTs as the target template. On the brain-extracted T1w, brain tissue segmentation of white matter, gray matter, and CSF was done with ‘fast’ (FSL 6.0.5.1:57b01774; RRID: SCR_002823; 49). ‘recon-all’ (in OASIS-3: *FreeSurfer* 6.0.1; in ADNI: *FreeSurfer* 7.2.0; RRID: SCR_001847; 50) was used for brain surface reconstruction. The previously estimated brain mask was refined with a custom variation of the method to reconcile segmentations of the cortical gray matter of Mindboggle derived from ANTs and *FreeSurfer* (RRID: SCR_002438; 51). Volumetric values were normalized by estimated intracranial volume, given by the volume distortion resulting from the affine registration from the native to the MNI305 standard space, to account for head-size effects.

b. Supplementary Tables

The *R^2^*, *MSE*, and *MAE* values for each of the 1000 splits of the models predicting continuous cognitive decline within datasets and all the features’ permutation importance values for each of the 1000 splits are available on [OSF](https://osf.io/up65f/?view_only=23ec3db71e814d048bb8461b24ad2f87).

##### Table 1

Overview of All the Included Features.

| Modality (# features) | Group (# features) | Test/Variables | Information |
| --- | --- | --- | --- |
| Non-brain (48) | Demographic information (3) | Age |  |
|  |  | Sex |  |
|  |  | Education |  |
|  | Clinical scores (23) | Mini-Mental State Examination (MMSE) | Sum score |
|  |  | Clinical Dementia Rating Scale Sum of Boxes (CDR-SOB) | 6 items; Global score; SOB score |
|  |  | Functional Activities Questionnaire (FAQ) | 10 items; Sum score |
|  |  | Neuropsychiatric Inventory Questionnaire (NPI-Q) | Symptom presence; Severity sum scores |
|  |  | Geriatric Depression Scale (GDS) | Sum score |
|  | Neuropsychological scores (8) | Wechsler Memory Scale-Revised (WMS-R) | Logical memory (LOGIMEM, MEMUNITS, MEMTIME); |
|  |  | Word fluency | Animals |
|  |  | Trail Making Test (TMT) | Part A (TRAIL A);  Part B (TRAIL B);  TRAILBnorm = TRAIL B / TRAIL A |
|  |  | Boston Naming Test (BNT) |  |
|  | Apolipoprotein E (APOE) (3) | Ε2 allele count |  |
|  |  | Ε3 allele count |  |
|  |  | Ε4 allele count |  |
|  | Cognitive Diagnosis (1) | Diagnosis | Healthy control, mild cognitive impairment, or dementia |
|  | Health information (9) | Cardio- and cerebrovascular health |  |
|  |  | Diabetes |  |
|  |  | Hypercholesterolemia |  |
|  | No. sessions before baseline (1) | No. sessions before baseline |  |
| Structural (35) | Regional grey matter volume (14) | Accumbens | Left and right |
|  |  | Amygdala | Left and right |
|  |  | Caudate | Left and right |
|  |  | Hippocampus | Left and right |
|  |  | Pallidum | Left and right |
|  |  | Putamen | Left and right |
|  |  | Thalamus | Left and right |
|  | Global measurements (21) | Total gray matter volume |  |
|  |  | Total subcortical gray matter volume |  |
|  |  | Lateral ventricles | Left and right |
|  |  | 3^rd^ ventricles | Left and right |
|  |  | 4^th^ ventricles | Left and right |
|  |  | Mean cortical thickness | Left and right |
|  |  | Total cortical volume | Left and right |
|  |  | Cerebral white matter volume | Left and right |
|  |  | Cerebellar white matter volume | Left and right |
|  |  | Cerebellar cortical volume | Left and right |
|  |  | Corpus callosum | Anterior, mid-anterior, central, mid-posterior and posterior |

*Note*. The following non-brain features were omitted from Vieira and colleagues [41] due to excess prevalence of missing values (above 25%): TMT Trail A (RR & LI), TMT Trail B (RR & LI), Word fluency (Vegetables), WMS-R Digit span (DIGIF, DIGIFLEN, DIGIB, DIGIBLEN), Wechsler Adult Intelligence Scale-Revised (WAIS-R) Digit symbol, Family history of dementia (diagnosis of dementia of mother, father and number of siblings diagnosed with dementia), smoking habits (has smoked within the last 30 days, smoked more than 100 cigarettes in their lifetime, total number of years smoking, the average number of cigarette packs smoked per day).

##### Table 2

Details on MRI Acquisition in OASIS-3 and ADNI.

| Dataset | | Voxel-size (mm × mm × mm) | TR (ms) | TE (ms) | Subjects |
| --- | --- | --- | --- | --- | --- |
|  | Scanner |  |  |  |  |
| OASIS-3 | |  |  |  |  |
|  | Biograph mMR | 1 × 1 × 1 | 2400 | 2.1 | 2 |
|  |  | 1 × 1.05 × 1.05 | 2300 | 3.0 | 42 |
|  | TrioTim | 1 × 1 × 1 | 2400 | 3.1 | 30 |
|  |  | 1 × 1.02 × 1.02 | 2400 | 3.1 | 1 |
|  |  | 1 × 1 × 1 | 2400 | 3.2 | 590 |
|  | Missing | 1 × 1 × 1 |  |  | 1 |
|  |  |  |  |  |  |
| ADNI | |  |  |  |  |
|  | Achieva | 1 × 1 × 1 |  | 2.9 | 24 |
|  |  | 1.2 × 1 × 1 |  | 3.1 | 120 |
|  |  | 1.2 × 1 × 1 |  | 3.2 | 19 |
|  |  | 1.2 × 1 × 1 |  | 3.3 | 13 |
|  | Achieva dStream | 1 × 1 × 1 |  | 2.9 | 44 |
|  | Allegra | 1.2 × 1 × 1 | 2300 | 2.9 | 29 |
|  | Biograph_mMR | 1 × 1 × 1 | 2300 | 3 | 6 |
|  | DISCOVERY MR750 | 1 × 1 × 1 |  | 3.1 | 62 |
|  |  | 1 × 1 × 1 |  | 3.2 | 1 |
|  |  | 1.2 × 1.02 × 1.02 |  | 3 | 97 |
|  |  | 1.2 × 1.02 × 1.02 |  | 3.2 | 11 |
|  |  | 1.2 × 1.05 × 1.05 |  | 3 | 48 |
|  | DISCOVERY MR750w | 1 × 1 × 1 |  | 3.1 | 29 |
|  |  | 1.2 × 1.02 × 1.02 |  | 3.1 | 10 |
|  |  | 1.2 × 1.05 × 1.05 |  | 3.1 | 1 |
|  | GEMINI | 1.2 × 1 × 1 |  | 3.1 | 15 |
|  | GENESIS_SIGNA | 1.2 × 1.02 × 1.02 |  | 3.1 | 6 |
|  | Ingenia | 1 × 1 × 1 |  | 2.9 | 51 |
|  |  | 1 × 1 × 1 |  | 3 | 2 |
|  |  | 1.2 × 1 × 1 |  | 3.2 | 18 |
|  |  | 1.2 × 1.05 × 1.05 |  | 3.1 | 1 |
|  | Ingenuity | 1.2 × 1 × 1 |  | 3.1 | 3 |
|  | Intera | 1 × 1 × 1 |  | 2.9 | 2 |
|  |  | 1.2 × 1 × 1 |  | 3.1 | 13 |
|  |  | 1.2 × 1 × 1 |  | 3.2 | 52 |
|  |  | 1.2 × 1 × 1 |  | 3.3 | 7 |
|  | Intera Achieva | 1.2 × 1 × 1 |  | 3.3 | 1 |
|  | Prisma | 1 × 1 × 1 | 2300 | 3 | 77 |
|  |  | 1.2 × 1.05 × 1.05 | 2300 | 3 | 9 |
|  | Prisma_fit | 1 × 1 × 1 | 2300 | 3 | 195 |
|  |  | 1.2 × 1 × 1 | 2300 | 3 | 1 |
|  |  | 1.2 × 1.05 × 1.05 | 2300 | 3 | 30 |
|  |  | 1.2 × 1.25 × 1.25 | 1800 | 2.5 | 1 |
|  | SIGNA EXCITE | 1.2 × 1.02 × 1.02 |  | 2.8 | 8 |
|  |  | 1.2 × 1.02 × 1.02 |  | 3 | 12 |
|  | SIGNA HDx | 1.2 × 1.02 × 1.02 |  | 2.8 | 13 |
|  | SIGNA Premier | 1 × 1 × 1 |  | 2.9 | 8 |
|  |  | 1 × 1 × 1 |  | 3 | 4 |
|  |  | 1.3 × 1.05 × 1.05 |  | 2.9 | 1 |
|  | SIGNA UHP | 1 × 1 × 1 |  | 3 | 2 |
|  | Signa HDxt | 1.2 × 1.02 × 1.02 |  | 2.8 | 109 |
|  |  | 1.2 × 1.02 × 1.02 |  | 3 | 8 |
|  |  | 1.2 × 1.05 × 1.05 |  | 2.8 | 8 |
|  |  | 1.2 × 1.05 × 1.05 |  | 3 | 2 |
|  | Skyra | 1 × 1 × 1 | 2300 | 3 | 41 |
|  |  | 1.2 × 1 × 1 | 2300 | 3 | 76 |
|  |  | 1.2 × 1.02 × 1.02 | 2300 | 3 | 1 |
|  |  | 1.2 × 1.05 × 1.05 | 2300 | 3 | 32 |
|  | Skyra_fit | 1 × 1 × 1 | 2300 | 3 | 2 |
|  | Trio | 1.2 × 1 × 1 | 2300 | 2.9 | 60 |
|  |  | 1.2 × 1 × 1 | 2300 | 3 | 1 |
|  | TrioTim | 1 × 1 × 1 | 2300 | 3 | 41 |
|  |  | 1.2 × 1 × 1 | 2300 | 2.9 | 19 |
|  |  | 1.2 × 1 × 1 | 2300 | 3 | 335 |
|  |  | 1.2 × 1.02 × 1.02 | 2300 | 2.9 | 1 |
|  |  | 1.2 × 1.02 × 1.02 | 2300 | 3 | 1 |
|  |  | 1.2 × 1.05 × 1.05 | 2300 | 3 | 3 |
|  | Verio | 1 × 1 × 1 | 2300 | 3 | 58 |
|  |  | 1.2 × 0.98 × 0.98 | 2300 | 3 | 1 |
|  |  | 1.2 × 1 × 1 | 2000 | 3 | 1 |
|  |  | 1.2 × 1 × 1 | 2300 | 3 | 151 |
|  |  | 1.2 × 1.05 × 1.05 | 2300 | 3 | 2 |
|  |  | 1.2 × 1.09 × 1.09 | 2300 | 2.9 | 2 |

*Note*. Details on MRI acquisition from the OASIS-3 dataset are based on Vieira et al. (2022). Empty cells mean that data is missing. TR = repetition time; TE = echo time.

##### Table 3

Comparisons of Absolute Errors for Different Subgroups in Predictions Across Datasets.

| Dataset | Modality: (1) vs. (2) | CDR-SOB | | |  | MMSE | | |
| --- | --- | --- | --- | --- | --- | --- | --- | --- |
|  |  | *p* | *MAE* (1) | *MAE* (2) |  | *p* | *MAE* (1) | *MAE* (2) |
| OASIS-3 → ADNI | HC vs. MCI and AD | < .001 | 0.16 | 0.62 |  | < .001 | 0.31 | 1.1 |
|  | MCI vs. HC and AD | < .001 | 0.47 | 0.44 |  | < .001 | 0.77 | 0.81 |
|  | AD vs. HC and MCI | < .001 | 1.2 | 0.33 |  | < .001 | 2.1 | 0.57 |
|  | HC vs. MCI | < .001 | 0.16 | 0.47 |  | < .001 | 0.31 | 0.77 |
|  | HC vs. AD | < .001 | 0.16 | 1.2 |  | < .001 | 0.31 | 2.1 |
|  | MCI vs. AD | < .001 | 0.47 | 1.2 |  | < .001 | 0.77 | 2.1 |
|  | Female vs. Male | < .001 | 0.4 | 0.50 |  | < .001 | 0.73 | 0.84 |
|  | APOE E4: 0 vs. 1 and 2 | < .001 | 0.38 | 0.54 |  | < .001 | 0.66 | 0.95 |
|  | APOE E4: 1 vs. 0 and 2 | .472 | 0.49 | 0.43 |  | .014 | 0.88 | 0.74 |
|  | APOE E4: 2 vs. 0 and 1 | < .001 | 0.71 | 0.42 |  | < .001 | 1.2 | 0.74 |
|  | APOE E4: 0 vs. 1 | .014 | 0.38 | 0.49 |  | < .001 | 0.66 | 0.88 |
|  | APOE E4: 0 vs. 2 | < .001 | 0.38 | 0.71 |  | < .001 | 0.66 | 1.2 |
|  | APOE E4: 1 vs. 2 | < .001 | 0.49 | 0.71 |  | < .001 | 0.88 | 1.2 |
|  | Age: < 65 vs. ≥ 65 years | .838 | 0.41 | 0.46 |  | .419 | 0.69 | 0.81 |
|  | Age: < 70 vs. ≥ 70 years | < .001 | 0.38 | 0.50 |  | < .001 | 0.67 | 0.87 |
|  | Age: < 75 vs. ≥ 75 years | < .001 | 0.41 | 0.53 |  | < .001 | 0.75 | 0.87 |
|  | Age: < 80 vs. ≥ 80 years | < .001 | 0.43 | 0.56 |  | < .001 | 0.76 | 0.98 |
| ADNI → OASIS-3 | HC vs. MCI and AD | < .001 | 0.19 | 0.69 |  | < .001 | 0.40 | 0.99 |
|  | MCI vs. HC and AD | .014 | 0.66 | 0.30 |  | > .999 | 0.58 | 0.53 |
|  | AD vs. HC and MCI | < .001 | 0.74 | 0.21 |  | < .001 | 1.1 | 0.42 |
|  | HC vs. MCI | < .001 | 0.19 | 0.66 |  | .939 | 0.40 | 0.58 |
|  | HC vs. AD | < .001 | 0.19 | 0.74 |  | < .001 | 0.40 | 1.1 |
|  | MCI vs. AD | .841 | 0.66 | 0.74 |  | .731 | 0.58 | 1.1 |
|  | Female vs. Male | < .001 | 0.27 | 0.34 |  | < .001 | 0.48 | 0.60 |
|  | APOE E4: 0 vs. 1 and 2 | .009 | 0.26 | 0.37 |  | .398 | 0.47 | 0.64 |
|  | APOE E4: 1 vs. 0 and 2 | .087 | 0.37 | 0.27 |  | .758 | 0.63 | 0.49 |
|  | APOE E4: 2 vs. 0 and 1 | .051 | 0.40 | 0.30 |  | .939 | 0.70 | 0.52 |
|  | APOE E4: 0 vs. 1 | .048 | 0.26 | 0.37 |  | .670 | 0.47 | 0.63 |
|  | APOE E4: 0 vs. 2 | .015 | 0.26 | 0.40 |  | .758 | 0.47 | 0.70 |
|  | APOE E4: 1 vs. 2 | .336 | 0.37 | 0.40 |  | > .999 | 0.63 | 0.70 |
|  | Age: < 65 vs. ≥ 65 years | < .001 | 0.17 | 0.33 |  | .015 | 0.36 | 0.57 |
|  | Age: < 70 vs. ≥ 70 years | < .001 | 0.21 | 0.38 |  | .018 | 0.43 | 0.62 |
|  | Age: < 75 vs. ≥ 75 years | < .001 | 0.23 | 0.46 |  | .006 | 0.46 | 0.70 |
|  | Age: < 80 vs. ≥ 80 years | < .001 | 0.27 | 0.51 |  | .024 | 0.49 | 0.75 |

*Note*. The *p* values of comparisons of absolute errors for different subgroups in predictions across datasets are displayed. (1) refers to the left side of the comparison, (2) refers to the right side of the comparison. Two-sided Mann-Whitney U tests (α = .05) for comparison of absolute errors were performed. *p* values were corrected for multiple comparisons using the Holm-Bonferroni method. OASIS-3 → ADNI = model trained on OASIS-3 and tested on ADNI; ADNI → OASIS-3 = model trained on ADNI and tested on OASIS-3. HC = healthy control; MCI = mild cognitive impairment, AD = Alzheimer’s disease or another form of dementia; APOE = apolipoprotein E; *MAE* = mean absolute error.

##### Table 4

Permutation Importance of All Features of OASIS-3 and ADNI.

|  | OASIS-3 | | | |  | | ADNI | | | |
| --- | --- | --- | --- | --- | --- | --- | --- | --- | --- | --- |
|  | Feature | | PI | |  | | Feature | | PI | |
|  | mmse_mmse | | 0.072  (0.069, 0.075) | |  | | TMTB_TRAILB | | 0.040  (0.038, 0.042) | |
|  | WMSlm_MEMUNITS | | 0.071  (0.067, 0.075) | |  | | mmse_mmse | | 0.031  (0.030, 0.032) | |
|  | faq_faqSum | | 0.053  (0.049, 0.057) | |  | | WMSlm_MEMUNITS | | 0.030  (0.028, 0.031) | |
|  | Vol. of Left-Hippocampus | | 0.027  (0.026, 0.029) | |  | | faq_BILLS | | 0.013  (0.012, 0.014) | |
|  | WMSlm_LOGIMEM | | 0.018  (0.016, 0.019) | |  | | WMSlm_LOGIMEM | | 0.012  (0.011, 0.013) | |
|  | TMTB_TRAILB | | 0.013  (0.012, 0.014) | |  | | TMTA_TRAILA | | 0.0090  (0.0075, 0.011) | |
|  | cdr_sob | | 0.0068  (0.0050, 0.0086) | |  | | faq_faqSum | | 0.0075  (0.0061, 0.0088) | |
|  | Vol. of Left-Amygdala | | 0.0057  (0.0043, 0.0070) | |  | | BOSTON_BOSTON | | 0.0062  (0.0053, 0.0071) | |
|  | WF_ANIMALS | | 0.0050  (0.0041, 0.0058) | |  | | WF_ANIMALS | | 0.0061  (0.0043, 0.0079) | |
|  | Vol. of Right-Accumbens-area | | 0.0045  (0.0039, 0.0051) | |  | | lh_MeanThickness_thickness | | 0.0061  (0.0055, 0.0066) | |
|  | Vol. of CC_Central | | 0.0043  (0.0035, 0.0050) | |  | | npiq_npiqSevSum | | 0.0048  (0.0042, 0.0054) | |
|  | Vol. of Right-Amygdala | | 0.0040  (0.0032, 0.0049) | |  | | demo_age | | 0.0039  (0.0035, 0.0044) | |
|  | TMTB_TRAILBnorm | | 0.0037  (0.0032, 0.0042) | |  | | Vol. of Right-Hippocampus | | 0.0037  (0.0034, 0.0041) | |
|  | Vol. of Right-Hippocampus | | 0.0037  (0.0029, 0.0044) | |  | | Vol. of 4th-Ventricle | | 0.0036  (0.0032, 0.0040) | |
|  | Vol. of Right-Thalamus-Proper | | 0.0032 (0.0026, 0.0039) | |  | | faq_TAXES | | 0.0030 (0.0024, 0.0037) | |
|  | faq_REMDATES | | 0.0031  (0.0024, 0.0037) | |  | | Vol. of Left-Amygdala | | 0.0027  (0.0023, 0.0032) | |
|  | Vol. of Left-Pallidum | | 0.0025  (0.0021, 0.0030) | |  | | TMTB_TRAILBnorm | | 0.0024  (0.0018, 0.0030) | |
|  | lhCerebralWhiteMatterVol | | 0.0021  (0.0014, 0.0027) | |  | | Vol. of 3rd-Ventricle | | 0.0021  (0.0016, 0.0026) | |
|  | Vol. of Left-Cerebellum-White-Matter | | 0.0019  (0.0015, 0.0023) | |  | | lhCortexVol | | 0.0015  (0.00095, 0.0021) | |
|  | npiq_npiqPresSum | | 0.0018  (0.0015, 0.0022) | |  | | Vol. of Left-Accumbens-area | | 0.0014  (0.00086, 0.0019) | |
|  | Vol. of Left-Accumbens-area | | 0.0018  (0.0012, 0.0024) | |  | | faq_EVENTS | | 0.0013  (0.00091, 0.0017) | |
|  | WMSlm_MEMTIME | | 0.0014  (0.0011, 0.0018) | |  | | rhCortexVol | | 0.0013  (0.00057, 0.0020) | |
|  | cdr_homehobb | | 0.0014  (0.00077, 0.0020) | |  | | cdr_sob | | 0.0012  (0.00066, 0.0018) | |
|  | cvasc_HYPERTEN | | 0.0012  (0.00093, 0.0015) | |  | | cdr_memory | | 0.0012  (0.00072, 0.0017) | |
|  | Vol. of Right-Cerebellum-White-Matter | | 0.0012  (0.00072, 0.0016) | |  | | Vol. of Left-Hippocampus | | 0.0012  (0.00094, 0.0014) | |
|  | cdr_commun | | 0.00096  (0.00061, 0.0013) | |  | | Vol. of Right-Amygdala | | 0.00097  (0.00066, 0.0013) | |
|  | npiq_npiqSevSum | | 0.00073  (0.00042, 0.0010) | |  | | Vol. of Right-Accumbens-area | | 0.00092  (0.00056, 0.0013) | |
|  | Vol. of 4th-Ventricle | | 0.00071  (0.00036, 0.0011) | |  | | Vol. of Left-Pallidum | | 0.00067  (0.00041, 0.00094) | |
|  | TMTA_TRAILA | | 0.00065  (0.00018, 0.0011) | |  | | faq_REMDATES | | 0.00054  (0.00013, 0.00095) | |
|  | apoe_e4count | | 0.00058 (0.00040, 0.00076) | |  | | WMSlm_MEMTIME | | 0.00047 (0.00025, 0.00069) | |
|  | Vol. of Right-Putamen | | 0.00053 (0.00019, 0.00087) | |  | | cvasc_HYPERTEN | | 0.00043 (0.00030, 0.00056) | |
|  | cdr_cdrGlobal | | 0.00051  (0.00013, 0.00089) | |  | | apoe_e4count | | 0.00040  (0.00027, 0.00053) | |
|  | Vol. of Right-Lateral-Ventricle | | 0.00048  (0.00013, 0.00082) | |  | | Vol. of CC_Central | | 0.00037  (0.00015, 0.00060) | |
|  | hypercho_HYPERCHO | | 0.00041  (0.00020, 0.00063) | |  | | rhCerebralWhiteMatterVol | | 0.00031  (-0.000039, 0.00066) | |
|  | Vol. of Left-Putamen | | 0.00038  (-0.000067, 0.00083) | |  | | Vol. of Left-Thalamus-Proper | | 0.00031  (0.000036, 0.00058) | |
|  | Vol. of SubCortGrayVol | | 0.00033  (0.000049, 0.00062) | |  | | npiq_npiqPresSum | | 0.00027  (-0.000074, 0.00062) | |
|  | Vol. of CC_Mid_Anterior | | 0.00031  (0.00000011, 0.00062) | |  | | Vol. of SubCortGrayVol | | 0.00021  (-0.0000016, 0.00041) | |
|  | diag | | 0.00019  (-0.00064, 0.0010) | |  | | rh_MeanThickness_thickness | | 0.00019  (-0.000087, 0.00046) | |
|  | demo_sex | | 0.00015  (0.0000030, 0.00029) | |  | | Vol. of CC_Anterior | | 0.00016  (-0.000067, 0.00039) | |
|  | Vol. of CC_Anterior | | 0.000090  (-0.00025, 0.00043) | |  | | Vol. of CC_Mid_Posterior | | 0.00016  (-0.000064, 0.00039) | |
|  | diabetes_DIABETES | | 0.000065  (-0.000044, 0.00017) | |  | | demo_education | | 0.00016  (0.0000022, 0.00032) | |
|  | session_n | | 0.000032  (-0.00013, 0.00020) | |  | | faq_GAMES | | 0.00015  (-0.00021, 0.00051) | |
|  | Vol. of Left-Lateral-Ventricle | | 0.000031  (-0.00044, 0.00050) | |  | | lhCerebralWhiteMatterVol | | 0.00014  (-0.00012, 0.00040) | |
|  | cdr_judgment | | 0.000010 (-0.00024, 0.00026) | |  | | cvasc_CVAFIB | | 0.00013 (0.00010, 0.00015) | |
|  | apoe_e3count | | 0.0000041  (-0.00015, 0.00016) | |  | | demo_sex | | 0.00010  (-0.00000093, 0.00021) | |
|  | cdr_perscare | | -0.000028  (-0.00011, 0.000053) | |  | | apoe_e3count | | 0.000072  (-0.000033, 0.00018) | |
|  | cvasc_CVHATT | | -0.000051  (-0.00012, 0.000018) | |  | | Vol. of Left-Caudate | | 0.000059  (-0.00014, 0.00026) | |
|  | faq_BILLS | | -0.000077  (-0.00029, 0.00013) | |  | | cdr_orient | | 0.000056  (-0.00012, 0.00023) | |
|  | cvasc_CVOTHR | | -0.000077  (-0.00016, 0.0000086) | |  | | session_n | | 0.000040  (0.0000054, 0.000075) | |
|  | faq_STOVE | | -0.000080  (-0.00017, 0.000014) | |  | | Vol. of Right-Thalamus-Proper | | 0.000038  (-0.00021, 0.00029) | |
|  | Vol. of Left-Caudate | | -0.00010  (-0.00042, 0.00021) | |  | | apoe_e2count | | 0.000024  (-0.000020, 0.000068) | |
|  | apoe_e2count | | -0.00010  (-0.00017, -0.000038) | |  | | Vol. of Right-Cerebellum-Cortex | | 0.0000031  (-0.00018, 0.00019) | |
|  | demo_education | | -0.00013  (-0.00065, 0.00040) | |  | | cvasc_CVHATT | | 0.00000025  (-0.0000044, 0.0000049) | |
|  | Vol. of Right-Pallidum | | -0.00016  (-0.00039, 0.000070) | |  | | cvasc_CBSTROKE | | -0.000016  (-0.000032, 0.00000022) | |
|  | cvasc_CVAFIB | | -0.00017 (-0.00024, -0.000099) | |  | | cdr_judgment | | -0.000019 (-0.00020, 0.00016) | |
|  | cvasc_CVANGIO | | -0.00018  (-0.00030,  -0.000051) | |  | | cvasc_CBTIA | | -0.000030  (-0.000053,  -0.0000081) | |
|  | Vol. of Left-Thalamus-Proper | | -0.00018  (-0.00041, 0.000050) | |  | | cdr_homehobb | | -0.000032  (-0.00036, 0.00030) | |
|  | Vol. of Left-Cerebellum-Cortex | | -0.00021  (-0.00051, 0.000076) | |  | | Vol. of Left-Cerebellum-White-Matter | | -0.000033  (-0.00033, 0.00026) | |
| Vol. of 3rd-Ventricle | | -0.00022  (-0.00054, 0.000095) | |  | | cdr_cdrGlobal | | -0.000034  (-0.00020, 0.00013) | |  |
| faq_GAMES | | -0.00023  (-0.00047, 0.0000058) | |  | | cdr_perscare | | -0.000045  (-0.00014, 0.000053) | |  |
| Vol. of CC_Mid_Posterior | | -0.00026  (-0.00092, 0.00039) | |  | | hypercho_HYPERCHO | | -0.000056  (-0.00013, 0.000021) | |  |
| faq_MEALPREP | | -0.00028  (-0.00046,  -0.00011) | |  | | diabetes_DIABETES | | -0.000064  (-0.00012,  -0.0000074) | |  |
| Vol. of Right-Cerebellum-Cortex | | -0.00030  (-0.00054,  -0.000055) | |  | | Vol. of Left-Cerebellum-Cortex | | -0.00011  (-0.00029, 0.000073) | |  |
| faq_PAYATTN | | -0.00033  (-0.00052,  -0.00014) | |  | | faq_PAYATTN | | -0.00012  (-0.00048, 0.00024) | |  |
| faq_SHOPPING | | -0.00034  (-0.00053,  -0.00015) | |  | | diag | | -0.00012  (-0.00040, 0.00016) | |  |
| faq_TRAVEL | | -0.00042  (-0.00072,  -0.00011) | |  | | cdr_commun | | -0.00017  (-0.00037, 0.000038) | |  |
| cvasc_CBSTROKE | | -0.00052  (-0.00063,  -0.00041) | |  | | cvasc_CVOTHR | | -0.00019  (-0.00025, -0.00013) | |  |
| faq_TAXES | | -0.00053  (-0.00089,  -0.00017) | |  | | cvasc_CVANGIO | | -0.00019  (-0.00029,  -0.000092) | |  |
| gds_gdsSum | | -0.00054  (-0.00078,  -0.00030) | |  | | faq_MEALPREP | | -0.00020  (-0.00058, 0.00018) | |  |
| rhCortexVol | | -0.00058  (-0.0011,  -0.0000075) | |  | | Vol. of Right-Pallidum | | -0.00022  (-0.00042,  -0.000015) | |  |
| cvasc_CBTIA | | -0.00061  (-0.00087,  -0.00035) | |  | | gds_gdsSum | | -0.00032  (-0.00047, -0.00017) | |  |
| cdr_orient | | -0.00065  (-0.00093,  -0.00038) | |  | | Vol. of Left-Putamen | | -0.00034  (-0.00053, -0.00014) | |  |
| rhCerebralWhiteMatterVol | | -0.00068  (-0.0011, -0.00027) | |  | | Vol. of Right-Cerebellum-White-Matter | | -0.00036  (-0.00060, -0.00012) | |  |
| demo_age | | -0.00070  (-0.0012, -0.00020) | |  | | Vol. of Right-Putamen | | -0.00043  (-0.00065, -0.00020) | |  |
| Vol. of Right-Caudate | | -0.00079  (-0.0011, -0.00048) | |  | | Vol. of CC_Mid_Anterior | | | -0.00046  (-0.00069, -0.00022) | |
| faq_EVENTS | | -0.00084  (-0.0011, -0.00060) | |  | | Vol. of Right-Caudate | | | -0.00053  (-0.00071, -0.00035) | |
| lh_MeanThickness_thickness | | -0.00087  (-0.0015, -0.00024) | |  | | Vol. of TotalGrayVol | | | -0.00061  (-0.00093, -0.00030) | |
| Vol. of TotalGrayVol | | -0.00095 (-0.0016, -0.00030) | |  | | faq_STOVE | | | -0.00066 (-0.00087, -0.00045) | |
| Vol. of CC_Posterior | | -0.0010  (-0.0012, -0.00082) | |  | | Vol. of Left-Lateral-Ventricle | | | -0.00078  (-0.0010, -0.00056) | |
| cdr_memory | | -0.0011  (-0.0014, -0.00070) | |  | | faq_SHOPPING | | | -0.00089  (-0.0012, -0.00056) | |
| rh_MeanThickness_thickness | | -0.0017  (-0.0021, -0.0014) | |  | | Vol. of CC_Posterior | | | -0.00098  (-0.0012, -0.00078) | |
| BOSTON_BOSTON | | -0.0022  (-0.0028, -0.0016) | |  | | faq_TRAVEL | | | -0.0011  (-0.0014, -0.00074) | |
| lhCortexVol | | -0.0033  (-0.0042, -0.0024) | |  | | Vol. of Right-Lateral-Ventricle | | | -0.0012  (-0.0015, -0.00086) | |

*Note*. Permutation importance of all input features of the models including non-brain data and structural MRI data for prediction within dataset, ordered by importance. PI = median permutation importance across 1000 splits.

##### Table 5

Overview of Model Performance Including Post hoc Analyses With Matched Samples, Late Fusion Approaches, and Varying Numbers of Top Features.

| Dataset | Modality | CDR-SOB | | |  | MMSE | | |
| --- | --- | --- | --- | --- | --- | --- | --- | --- |
|  |  | *R^2^* | *MSE* | *MAE* |  | *R^2^* | *MSE* | *MAE* |
| OASIS-3  → ADNI | non-brain | .30 | .65 | .50 |  | .25 | 1.99 | .81 |
|  | structural MRI | .17 | .77 | .49 |  | .10 | 2.37 | .89 |
|  | combined | .35 | .60 | .45 |  | .26 | 1.95 | .79 |
|  | combined *matched* | .22 | .36 | .38 |  | .18 | .84 | .56 |
|  | late fusion *weighted* | .32 | .64 | .47 |  | .25 | 1.98 | .79 |
|  | late fusion *averaged* | .30 | .65 | .46 |  | .23 | 2.03 | .80 |
|  | top 15 | .34 | .61 | .42 |  | .26 | 1.96 | .80 |
|  | top 15 *matched* | .27 | .34 | .33 |  | .16 | .87 | .57 |
|  | top 5 | .25 | .70 | .47 |  | .17 | 2.18 | .86 |
|  | top 10 | .35 | .60 | .43 |  | .29 | 1.88 | .79 |
|  | top 20 | .35 | .61 | .43 |  | .26 | 1.95 | .80 |
| ADNI → OASIS-3 | non-brain | .28 | .27 | .29 |  | .19 | .71 | .48 |
|  | structural MRI | −.02 | .38 | .41 |  | −.15 | 1.00 | .68 |
|  | combined | .31 | .26 | .30 |  | .18 | .72 | .53 |
|  | combined *matched* | .29 | .42 | .42 |  | .29 | 1.1 | .68 |
|  | late fusion *weighted* | .28 | .27 | .29 |  | .19 | .71 | .48 |
|  | late fusion *averaged* | .33 | .32 | .25 |  | .24 | .66 | .51 |
|  | top 15 | .31 | .26 | .27 |  | .18 | .71 | .47 |
|  | top 15 *matched* | .29 | .42 | .39 |  | .25 | 1.2 | .65 |
|  | top 5 | .13 | .32 | .32 |  | .03 | .84 | .54 |
|  | top 10 | .25 | .28 | .28 |  | .16 | .73 | .49 |
|  | top 20 | .31 | .26 | .27 |  | .18 | .72 | .48 |

*Note*. This Table is an extended and adapted version of Table 2, including the post hoc analyses with matched samples, late fusion approaches, and varying numbers of top features, excluding the model comparisons. *matched* indicates that the distribution of the predictive targets (CDR-SOB and MMSE slope) of the test set was matched to that of the training set in predictions across datasets. In the late fusion strategy, separate models were trained on non-brain and structural MRI features independently. These models generate predictions over the target variables, which are then combined using either *R²*-weighted averaging (*weighted*; where each modality's contribution is weighted by its predictive performance) or simple averaging (*averaged*). A sensitivity analysis was conducted to assess the effect of the number of top features on model performance, varying the threshold across top-5, top-10, top-15, and top-20 features. OASIS-3 → ADNI = model trained on OASIS-3 and tested on ADNI; ADNI → OASIS-3 = model trained on ADNI and tested on OASIS-3; CDR-SOB = Clinical Dementia Rating Scale Sum of Boxes; MMSE = Mini-Mental State Examination; *R^2^* = median coefficient of determination across 1000 splits; *MSE* = mean squared error; *MAE* = mean absolute error.

##### Table 6

*Sign Mismatch Analysis per Diagnostic Subgroup: True vs. Predicted Slope Directions.*

|  | HC | MCI | AD | Total |
| --- | --- | --- | --- | --- |
| OASIS-3 → ADNI: CDR-SOB |  |  |  |  |
| Correct direction | 436 (96.0%) | 421 (69.4%) | 158 (89.8%) | 1015 |
| True improvement, predicted worsening | 16 (3.5%) | 182 (30.0%) | 18 (10.2%) | 216 |
| True worsening, predicted improvement | 2 (0.4%) | 4 (0.7%) | 0 (0.0%) | 6 |
| ADNI → OASIS-3: CDR-SOB |  |  |  |  |
| Correct direction | 495 (97.2%) | 8 (66.7%) | 91 (82.0%) | 594 |
| True improvement, predicted worsening | 13 (2.6%) | 4 (33.3%) | 20 (18.0%) | 37 |
| True worsening, predicted improvement | 1 (0.2%) | 0 (0.0%) | 0 (0.0%) | 1 |
| OASIS-3 → ADNI: MMSE |  |  |  |  |
| Correct direction | 359 (79.1%) | 466 (76.8%) | 139 (79.0%) | 964 |
| True improvement, predicted worsening | 46 (10.1%) | 118 (19.4%) | 37 (21.0%) | 201 |
| True worsening, predicted improvement | 49 (10.8%) | 23 (3.8%) | 0 (0.0%) | 72 |
| ADNI → OASIS-3: MMSE |  |  |  |  |
| Correct direction | 391 (76.8%) | 12 (100.0%) | 91 (82.0%) | 494 |
| True improvement, predicted worsening | 97 (19.1%) | 0 (0.0%) | 18 (16.2%) | 115 |
| True worsening, predicted improvement | 21 (4.1%) | 0 (0.0%) | 2 (1.8%) | 23 |

*Note*. The true and predicted values of CDR-SOB and MMSE change were derived from combined models predicting continuous cognitive decline across datasets. For the CDR-SOB, in which higher scores indicate more impairment, “true improvement, predicted worsening” means that the true slope had a negative sign, while the predicted slope had a positive sign. “True worsening, predicted improvement” means that the true slope had a positive sign, while the predicted slope had a negative sign. “Correct direction” indicates a sign match of the true and the predicted slopes. For the MMSE, in which lower scores indicate more impairment, “true improvement, predicted worsening” means that the true slope had a positive sign, while the predicted slope had a negative sign. “True worsening, predicted improvement” means that the true slope had a negative sign, while the predicted slope had a positive sign. “Correct direction” again indicates a sign match of the true and the predicted slopes. OASIS-3 → ADNI = model trained on OASIS-3 and tested on ADNI; ADNI → OASIS-3 = model trained on ADNI and tested on OASIS-3; CDR-SOB = Clinical Dementia Rating Scale Sum of Boxes; MMSE = Mini-Mental State Examination.

##### Table 7

*Sign Mismatch Analysis per Diagnosis Subgroup: True vs. Predicted Slope Directions.*

|  | Female | Male | Total |
| --- | --- | --- | --- |
| OASIS-3 → ADNI: CDR-SOB |  |  |  |
| Correct direction | 496 (83.4%) | 519 (80.8%) | 1015 |
| True improvement, predicted worsening | 96 (16.1%) | 120 (18.7%) | 216 |
| True worsening, predicted improvement | 3 (0.5%) | 3 (0.5%) | 6 |
| ADNI → OASIS-3: CDR-SOB |  |  |  |
| Correct direction | 341 (94.7%) | 271 (89.7%) | 612 |
| True improvement, predicted worsening | 18 (5.0%) | 31 (10.3%) | 49 |
| True worsening, predicted improvement | 1 (0.3%) | 0 (0.0%) | 1 |
| OASIS-3 → ADNI: MMSE |  |  |  |
| Correct direction | 477 (80.2%) | 487 (75.9%) | 1015 |
| True improvement, predicted worsening | 85 (14.3%) | 116 (18.1%) | 201 |
| True worsening, predicted improvement | 33 (5.5%) | 39 (6.1%) | 72 |
| ADNI → OASIS-3: MMSE |  |  |  |
| Correct direction | 292 (81.1%) | 228 (75.5%) | 520 |
| True improvement, predicted worsening | 55 (15.3%) | 63 (20.9%) | 118 |
| True worsening, predicted improvement | 13 (3.6%) | 11 (3.6%) | 24 |

*Note*. The true and predicted values of CDR-SOB and MMSE change were derived from combined models predicting continuous cognitive decline across datasets. For the CDR-SOB, in which higher scores indicate more impairment, “true improvement, predicted worsening” means that the true slope had a negative sign, while the predicted slope had a positive sign. “True worsening, predicted improvement” means that the true slope had a positive sign, while the predicted slope had a negative sign. “Correct direction” indicates a sign match of the true and the predicted slopes. For the MMSE, in which lower scores indicate more impairment, “true improvement, predicted worsening” means that the true slope had a positive sign, while the predicted slope had a negative sign. “True worsening, predicted improvement” means that the true slope had a negative sign, while the predicted slope had a positive sign. “Correct direction” again indicates a sign match of the true and the predicted slopes. OASIS-3 → ADNI = model trained on OASIS-3 and tested on ADNI; ADNI → OASIS-3 = model trained on ADNI and tested on OASIS-3; CDR-SOB = Clinical Dementia Rating Scale Sum of Boxes; MMSE = Mini-Mental State Examination.

##### Table 8

*Feature block permutation importance results.*

| Feature block | OASIS-3 PI | ADNI PI |
| --- | --- | --- |
| Neuropsychological tests | 0.176 (0.170, 0.182) | 0.210 (0.207, 0.214) |
| Clinical | 0.0729 (0.0697, 0.0761) | 0.0334 (0.0321, 0.0348) |
| Hippo-amygdala | 0.0683 (0.0655, 0.0711) | 0.0148 (0.0140, 0.0156) |
| FAQ | 0.0676 (0.0634, 0.0719) | 0.0658 (0.0636, 0.0681) |
| Striatum & pallidum | 0.0116 (0.0102, 0.0129) | 0.00179 (0.00102, 0.00256) |
| Corpus-callosum | 0.00600 (0.00463, 0.00736) | -0.000711 (-0.00120, -0.000225) |
| Cerebellar | 0.00349 (0.00260, 0.00438) | -0.000127 (-0.000596, 0.000343) |
| Thalamus | 0.00287 (0.00217, 0.00357) | 0.000441 (0.0000431, 0.000839) |
| Ventricles | 0.00133 (0.000598, 0.00205) | 0.00398 (0.00331, 0.00464) |
| APOE | 0.000552 (0.000298, 0.000806) | 0.000594 (0.000421, 0.000767) |
| Health | 0.000162 (-0.000360, 0.000685) | 0.0000834 (-0.000123, 0.000290) |
| Demographics | -0.000823 (-0.00153, -0.000118) | 0.00426 (0.00377, 0.00474) |
| Cerebral | -0.00183 (-0.00385, 0.000194) | 0.0185 (0.0170, 0.0200) |
| Language | -0.00218 (-0.00278, -0.00158) | 0.00602 (0.00511, 0.00693) |

*Note.* Feature block permutation-based importance results, ordered by the mean reduction in performance in OASIS-3. The procedure consists in permuting all features in each block simultaneously, maintaining the within-block covariance structure but breaking the between-block patterns. Neuropsychological tests: WMS, word fluency, TMT. Clinical: Session number, baseline diagnosis, CDR-SOB, MMSE, GDS, NPI-Q. Hippo-amygdala: left and right hemisphere hippocampal and amygdalar volumes. FAQ: FAQ. Striatum & pallidum: left and right hemisphere nucleus accumbens, caudate, globus pallidum and putamen volumes. Corpus-callosum: anterior, mid-anterior, central, mid-posterior and posterior corpus callosum volumes. Cerebellar: left and right hemisphere cerebellar white- and gray-matter volumes. Thalamus: left and right thalamus. Ventricles: left and right lateral, 3^rd^ and 4^th^ ventricles volumes. APOE: APOE ε2, ε3 and ε4 allele counts. Health: diabetes, hypercholesterolemia, stroke, transient ischemic attack, and cardiovascular conditions. Demographics: sex, age and education. Cerebral: hemispheric total volume of gray-matter, cerebral white matter, cortex and hemispheric mean cortical thickness. Language: Boston naming test.

c. Supplementary Figures

##### Figure 1

Inclusion of Subjects of the ADNI Dataset.

*.*

*Note*. The final ADNI dataset consisted of 1237 participants. T1w MRI = T1-weighted magnetic resonance imaging.

##### Figure 2

*Distribution of CDR-SOB and MMSE Change in OASIS-3 and ADNI.*

*Note*. Panels A and C: Distribution of observed cognitive decline measured via Clinical Dementia Rating Scale Sum of Boxes (CDR-SOB change) and Mini-Mental State Examination (MMSE change) in the OASIS-3 dataset. Panels B and D: Distribution of observed CDR-SOB change and MMSE change in the ADNI dataset. CDR-SOB change: Positive values indicate cognitive decline. MMSE change: Negative values indicate cognitive decline. The number and the dashed vertical line represent the mean cognitive change.

##### Figure 3

Distribution of R^2^ Across 1000 Splits in OASIS-3 Models.

*Note*. Distribution of *R^2^* (coefficient of determination) across 1000 splits for predicting cognitive decline measured via Clinical Dementia Rating Scale Sum of Boxes (CDR-SOB change) (in panels A, B and C) and Mini-Mental State Examination (MMSE change) (in panels D, E and F) when using different input modalities (non-brain; structural MRI; combined) in OASIS-3 data. The number and the dashed vertical line represent the median *R^2^* across 1000 splits.

##### Figure 4

Distribution of R^2^ Across 1000 Splits in ADNI Models.

*Note*. Distribution of *R^2^* (coefficient of determination) across 1000 splits for predicting cognitive decline measured via Clinical Dementia Rating Scale Sum of Boxes (CDR-SOB change) (in panels A, B and C) and Mini-Mental State Examination (MMSE change) (in panels D, E and F) when using different input modalities (non-brain; structural MRI; combined) in ADNI data. The number and the dashed vertical line represent the median *R^2^* across 1000 splits.

##### Figure 5

True Versus Predicted Values of CDR-SOB and MMSE Change for Subgroups Differentiated by Clinical Diagnoses in Predictions Across Datasets.

*Note*. Combined models included non-brain data and structural MRI data to predict continuous cognitive decline across datasets. The scatter plot shows true versus predicted values of CDR-SOB and MMSE change. CDR-SOB change: Positive values indicate cognitive decline. MMSE change: Negative values indicate cognitive decline. Each data point represents an individual participant of the respective test set. Data points are differentiated regarding the clinical diagnoses of the participants in the test set. HC = healthy control; MCI = mild cognitive impairment, AD = Alzheimer’s disease or another form of dementia; OASIS-3 → ADNI = model trained on OASIS-3 and tested on ADNI; ADNI → OASIS-3 = model trained on ADNI and tested on OASIS-3. 30 OASIS-3 participants with missing diagnoses are omitted in C and D.

##### Figure 6

Feature Importance for Predictions Across Datasets

*Note*. Permutation importance of the top 15 features of the combined models for prediction across dataset with 100 repetitions, ordered by average importance. Models simultaneously predicted the slopes of CDR-SOB and MMSE change.

##### Figure 7

Performance of single- vs multi-output models at different training set sizes

*Note*. To help assess the non-inferiority of the multi-output model, we additionally trained and tested single-output models in the prediction of CDR-SOB and MMSE slope across datasets. Multi-output and single-output models’ budgets are matched, i.e., 50 trees are estimated for each single-output model compared to 100 trees for the multi-output models. To assess the effect of training set size and determine whether model performance had reached a plateau, models were trained on subsets of the training data containing n=60,100,200,300, and 600 samples. This procedure was repeated 100 times using different random resamples.

1 References

[1]	Scheltens P, Strooper B de, Kivipelto M, Holstege H, Chételat G, Teunissen CE et al. Alzheimer's disease. Lancet 2021;397(10284):1577–90.

[2]	Altmann A, Tian L, Henderson VW, Greicius MD. Sex modifies the APOE-related risk of developing Alzheimer disease. Annals of neurology 2014;75(4):563–73.

[3]	Chêne G, Beiser A, Au R, Preis SR, Wolf PA, Dufouil C et al. Gender and incidence of dementia in the Framingham Heart Study from mid-adult life. Alzheimer's & Dementia 2015;11(3):310–20.

[4]	Caamaño-Isorna F, Corral M, Montes-Martínez A, Takkouche B. Education and dementia: a meta-analytic study. Neuroepidemiology 2006;26(4):226–32.

[5]	Rasmussen KL, Tybjærg-Hansen A, Nordestgaard BG, Frikke-Schmidt R. Absolute 10-year risk of dementia by age, sex and APOE genotype: a population-based cohort study. CMAJ Canadian Medical Association journal = journal de l'Association medicale canadienne 2018;190(35):E1033-E1041.

[6]	Morris JC. The Clinical Dementia Rating (CDR): current version and scoring rules. Neurology 1993;43(11):2412–4.

[7]	Folstein MF, Folstein SE, McHugh PR. “Mini-mental state”: a practical method for grading the cognitive state of patients for the clinician. Journal of Psychiatric Research 1975;12(3):189–98.

[8]	Jette AM, Davies AR, Cleary PD, Calkins DR, Rubenstein LV, Fink A et al. The Functional Status Questionnaire: reliability and validity when used in primary care. Journal of general internal medicine 1986;1(3):143–9.

[9]	Yesavage JA, Brink TL, Rose TL, Lum O, Huang V, Adey M et al. Development and validation of a geriatric depression screening scale: a preliminary report. Journal of Psychiatric Research 1982;17(1):37–49.

[10]	Kaufer DI, Cummings JL, Ketchel P, Smith V, MacMillan A, Shelley T et al. Validation of the NPI-Q, a brief clinical form of the Neuropsychiatric Inventory. The Journal of neuropsychiatry and clinical neurosciences 2000;12(2):233–9.

[11]	O'Bryant SE, Waring SC, Cullum CM, Hall J, Lacritz L, Massman PJ et al. Staging dementia using Clinical Dementia Rating Scale Sum of Boxes scores: a Texas Alzheimer's research consortium study. Arch Neurol 2008;65(8):1091–5.

[12]	Mitchell AJ. The Mini-Mental State Examination (MMSE): An Update on Its Diagnostic Validity for Cognitive Disorders: In: Larner AJ, editor. Cognitive Screening Instruments: A Practical Approach. London: Springer London; 2013, p. 15–46.

[13]	Teng E, Becker BW, Woo E, Knopman DS, Cummings JL, Lu PH. Utility of the functional activities questionnaire for distinguishing mild cognitive impairment from very mild Alzheimer disease. Alzheimer disease and associated disorders 2010;24(4):348–53.

[14]	Berezuk C, Khan M, Callahan BL, Ramirez J, Black SE, Zakzanis KK. Sex differences in risk factors that predict progression from mild cognitive impairment to Alzheimer's dementia. Journal of the International Neuropsychological Society 2023;29(4):360–8.

[15]	Livingston G, Huntley J, Sommerlad A, Ames D, Ballard C, Banerjee S et al. Dementia prevention, intervention, and care: 2020 report of the Lancet Commission. Lancet 2020;396(10248):413–46.

[16]	Acosta I, Borges G, Aguirre-Hernandez R, Sosa AL, Prince M. Neuropsychiatric symptoms as risk factors of dementia in a Mexican population: A 10/66 Dementia Research Group study. Alzheimer's & Dementia 2018;14(3):271–9.

[17]	Mallo SC, Valladares-Rodriguez S, Facal D, Lojo-Seoane C, Fernández-Iglesias MJ, Pereiro AX. Neuropsychiatric symptoms as predictors of conversion from MCI to dementia: a machine learning approach. International psychogeriatrics 2020;32(3):381–92.

[18]	Dolcet-Negre MM, Imaz Aguayo L, García-de-Eulate R, Martí-Andrés G, Fernández-Matarrubia M, Domínguez P et al. Predicting conversion from subjective cognitive decline to mild cognitive impairment and Alzheimer's disease dementia using ensemble machine learning. Journal of Alzheimer's Disease 2023;93(1):125–40.

[19]	Borod JC, Goodglass H, Kaplan E. Normative data on the boston diagnostic aphasia examination, parietal lobe battery, and the boston naming Test. Journal of Clinical Neuropsychology 1980;2(3):209–15.

[20]	Bucks RS. Trail-Making Test: In: Gellman MD, Turner JR, editors. Encyclopedia of behavioral medicine. New York, NY: Springer; 2013, p. 1986–7.

[21]	Elwood RW. The Wechsler Memory Scale-Revised: psychometric characteristics and clinical application. Neuropsychology review 1991;2(2):179–201.

[22]	Rabin LA, Barr WB, Burton LA. Assessment practices of clinical neuropsychologists in the United States and Canada: a survey of INS, NAN, and APA Division 40 members. Archives of clinical neuropsychology the official journal of the National Academy of Neuropsychologists 2005;20(1):33–65.

[23]	Williams BW, Mack W, Henderson VW. Boston Naming Test in Alzheimer's disease. Neuropsychologia 1989;27(8):1073–9.

[24]	Eckerström C, Olsson E, Bjerke M, Malmgren H, Edman A, Wallin A et al. A combination of neuropsychological, neuroimaging, and cerebrospinal fluid markers predicts conversion from mild cognitive impairment to dementia. Journal of Alzheimer's Disease 2013;36(3):421–31.

[25]	Bowie CR, Harvey PD. Administration and interpretation of the Trail Making Test. Nature protocols 2006;1(5):2277–81.

[26]	Thompson MD, Scott JG, Dickson SW, Schoenfeld JD, Ruwe WD, Adams RL. Clinical Utility of the Trail Making Test Practice Time. The Clinical neuropsychologist 1999;13(4):450–5.

[27]	Eckerström C, Olsson E, Klasson N, Berge J, Nordlund A, Bjerke M et al. Multimodal prediction of dementia with up to 10 years follow up: the Gothenburg MCI study. Journal of Alzheimer's Disease 2015;44(1):205–14.

[28]	Ewers M, Walsh C, Trojanowski JQ, Shaw LM, Petersen RC, Jack CR Jr. et al. Prediction of conversion from mild cognitive impairment to Alzheimer's disease dementia based upon biomarkers and neuropsychological test performance. Neurobiology of aging 2012;33(7):1203–14.

[29]	Edwin TH, Strand BH, Persson K, Engedal K, Selbæk G, Knapskog A-B. Trajectories and risk factors of dementia progression: a memory clinic cohort followed up to 3 years from diagnosis. International psychogeriatrics 2021;33(8):779–89.

[30]	Ghanavati E, Salehinejad MA, Nejati V, Nitsche MA. Differential role of prefrontal, temporal and parietal cortices in verbal and figural fluency: Implications for the supramodal contribution of executive functions. Scientific reports 2019;9(1):3700.

[31]	Whiteside DM, Kealey T, Semla M, Luu H, Rice L, Basso MR et al. Verbal Fluency: Language or Executive Function Measure? Applied neuropsychology. Adult 2016;23(1):29–34.

[32]	Fernaeus S-E, Ostberg P, Hellström A, Wahlund L-O. Cut the coda: early fluency intervals predict diagnoses. Cortex; a journal devoted to the study of the nervous system and behavior 2008;44(2):161–9.

[33]	Jacobs S, Mercuri G, Holtzer R. Assessing within-task verbal fluency performance: the utility of individual time intervals in predicting incident mild cognitive impairment. Neuropsychology, development, and cognition. Section B, Aging, neuropsychology and cognition 2021;28(5):733–47.

[34]	Derrer DS, Howieson DB, Mueller EA, Camicioli RM, Sexton G, Kaye JA. Memory testing in dementia: how much is enough? Journal of geriatric psychiatry and neurology 2001;14(1):1–6.

[35]	Wood ME, Xiong LY, Wong YY, Buckley RF, Swardfager W, Masellis M et al. Sex differences in associations between APOE ε2 and longitudinal cognitive decline. Alzheimer's & Dementia 2023;19(10):4651–61.

[36]	Singh PP, Singh M, Mastana SS. APOE distribution in world populations with new data from India and the UK. Annals of human biology 2006;33(3):279–308.

[37]	Mirza SS, Saeed U, Knight J, Ramirez J, Stuss DT, Keith J et al. APOE ε4, white matter hyperintensities, and cognition in Alzheimer and Lewy body dementia. Neurology 2019;93(19):e1807-e1819.

[38]	Petersen RC, Smith GE, Waring SC, Ivnik RJ, Tangalos EG, Kokmen E. Mild cognitive impairment: clinical characterization and outcome. Arch Neurol 1999;56(3):303–8.

[39]	Bhagwat N, Viviano JD, Voineskos AN, Chakravarty MM. Modeling and prediction of clinical symptom trajectories in Alzheimer's disease using longitudinal data. PLoS computational biology 2018;14(9):e1006376.

[40]	Korolev IO, Symonds LL, Bozoki AC. Predicting progression from mild cognitive impairment to Alzheimer's dementia using clinical, MRI, and plasma biomarkers via probabilistic pattern classification. PloS one 2016;11(2):e0138866.

[41]	Vieira BH, Liem F, Dadi K, Engemann DA, Gramfort A, Bellec P et al. Predicting future cognitive decline from non-brain and multimodal brain imaging data in healthy and pathological aging. Neurobiology of aging 2022;118:55–65.

[42]	Esteban O, Markiewicz CJ, Blair RW, Moodie CA, Isik AI, Erramuzpe A et al. fMRIPrep: a robust preprocessing pipeline for functional MRI. Nature methods 2019;16(1):111–6.

[43]	Esteban O, Markiewicz CJ, Blair RW, Poldrack RA, Gorgolewski KJ. sMRIPrep: Structural MRI PREProcessing workflows: Zenodo; 2023.

[44]	Esteban O, Markiewicz CJ, Burns CD, Goncalves M, Jarecka D, Ziegler E et al. nipy/nipype: 1.8.1: Zenodo; 2022.

[45]	Gorgolewski KJ, Burns CD, Madison C, Clark D, Halchenko YO, Waskom ML et al. Nipype: a flexible, lightweight and extensible neuroimaging data processing framework in python. Frontiers in Neuroinformatics 2011;5:13.

[46]	Gorgolewski KJ, Esteban O, Markiewicz CJ, Ziegler E, Ellis DG, Jarecka D et al. nipy/nipype: 1.2.0: Zenodo; 2019.

[47]	Tustison NJ, Avants BB, Cook PA, Zheng Y, Egan A, Yushkevich PA et al. N4ITK: improved N3 bias correction. IEEE transactions on medical imaging 2010;29(6):1310–20.

[48]	Avants BB, Epstein CL, Grossman M, Gee JC. Symmetric diffeomorphic image registration with cross-correlation: evaluating automated labeling of elderly and neurodegenerative brain. Medical image analysis 2008;12(1):26–41.

[49]	Zhang X-X, Tian Y, Wang Z-T, Ma Y-H, Tan L, Yu J-T. The Epidemiology of Alzheimer's Disease Modifiable Risk Factors and Prevention. The journal of prevention of Alzheimer's disease 2021;8(3):313–21.

[50]	Dale AM, Fischl B, Sereno MI. Cortical surface-based analysis. I. Segmentation and surface reconstruction. NeuroImage 1999;9(2):179–94.

[51]	Klein A, Ghosh SS, Bao FS, Giard J, Häme Y, Stavsky E et al. Mindboggling morphometry of human brains. PLoS computational biology 2017;13(2):e1005350.
